# Recent Developments in 3D Bio-Printing and Its Biomedical Applications

**DOI:** 10.3390/pharmaceutics15010255

**Published:** 2023-01-11

**Authors:** Humira Assad, Arvina Assad, Ashish Kumar

**Affiliations:** 1 Department of Chemistry, School of Chemical Engineering and Physical Sciences, Lovely Professional University, Punjab 144001, India; 2 Bibi Halima College of Nursing and Medical Technology, Srinagar 190010, India; 3 Nalanda College of Engineering, Department of Science and Technology, Government of Bihar, Patna 803108, India

**Keywords:** 3D bio-printing, biomaterials, bio-ink, additive manufacturing, scaffold, tissue engineering

## Abstract

The fast-developing field of 3D bio-printing has been extensively used to improve the usability and performance of scaffolds filled with cells. Over the last few decades, a variety of tissues and organs including skin, blood vessels, and hearts, etc., have all been produced in large quantities via 3D bio-printing. These tissues and organs are not only able to serve as building blocks for the ultimate goal of repair and regeneration, but they can also be utilized as in vitro models for pharmacokinetics, drug screening, and other purposes. To further 3D-printing uses in tissue engineering, research on novel, suitable biomaterials with quick cross-linking capabilities is a prerequisite. A wider variety of acceptable 3D-printed materials are still needed, as well as better printing resolution (particularly at the nanoscale range), speed, and biomaterial compatibility. The aim of this study is to provide expertise in the most prevalent and new biomaterials used in 3D bio-printing as well as an introduction to the associated approaches that are frequently considered by researchers. Furthermore, an effort has been made to convey the most pertinent implementations of 3D bio-printing processes, such as tissue regeneration, etc., by providing the most significant research together with a comprehensive list of material selection guidelines, constraints, and future prospects.

## 1. Introduction

A collection of cutting-edge processes known as additive manufacturing (AM) enable the direct fabrication of 3D (three-dimensional) physical items from computer-aided design (CAD) data [1,2]. Metals, ceramics, and thermoplastic polymers are presently manufactured using a wide variety of AM processes [3,4]. A number of AM methodologies have been developed, among which 3D printing was created and used for prototyping and modeling in order to fabricate complex geometrical, affordable, and simple-to-operate parts for various applications (such as in the aerospace and automotive industries) [5,6,7]. Owing to their simplicity and quickness, recent advancements in the therapeutic sector and the development of inventive biodegradable materials [8] have attracted attention. Complex structures and components can be created with these, surpassing a number of restrictions imposed by standard fabrication methods [9]. The regeneration of tissue is the present focus of intensive research, due to the fact that it is the main procedure associated with cell formation and organ reconstruction. Patients with injured organs may be prospects for organ transplantation, replacement, or repair based on the situation and the severity of the injury. Scientists are eager to uncover new solutions to make up for the organ deficit in these conditions. It has been suggested that the use of tissue engineering (TE) could help resuscitate lives and enhance lifestyle quality. Tissue engineering, which was first proposed in 1993 [10], aims to create usable substitutes for injured tissue by combining engineering and biological principles. The 3D bio-printing method is a tissue-engineering approach that is currently being studied because it shows efficient management over scaffold construction and cell dissemination. In the 3D bio-printing of scaffolds, a mixture of cells and biomaterials is frequently utilized as the printing predecessor. Three-dimensional bio-printing, which combines biology and 3D printing, is a state-of-the-art technology in AM and involves the layer-by-layer (LBL) accumulation of biomaterials on strata, in which they are inserted into suitable biomaterials [11]. In other words, the procedure involves printing and patterning the cells using an automated dispensing process on a base or tissue [12]. When compared to alternative assembly methods such as molding or the use of porous scaffolds, 3D bio-printing approaches have a printing resolution that is flexible and ranges from 10 to 10,000 mm [13,14]. In 3D bio-printing, tiny biomaterial, biochemical, and living-cell entities are precisely positioned alongside useful components to produce tissue-like architectures [15]. Three-dimensional bio-printing has enabled the on-demand “printing” of cells, tissues, and organs, which has greatly advanced the healthcare industry. These innovations may also help to cure a number of ailments in veterinary medicine, such as horse bone fractures and articular cartilage repair, or the creation of more precise disease models when combined with tissue engineering advancements [16]. Numerous studies on 3D bio-printing have been conducted utilizing a variety of techniques, including in situ skin printing, 3D tissue printing, and bio-printing with inkjet technology. Nevertheless, 3D bio-printing necessitates various technical techniques, i.e., biomimicry, autonomous self-assembly, and mini-tissue structural components, to create 3D frameworks with mechanical and biological characteristics appropriate for the accumulation of living cells and the reconstruction of tissue and organ activity [17]. As an additive manufacturing method, 3D bio-printing relies on the micrometer-scale layering of biomaterials, either encasing cells or subsequently packed with cells to build delicate constructs resembling tissue. For breast reconstructive surgery, Rocco et al. developed 3D additive produced poly(ε-caprolactone) scaffolds with various topologies and internal pore geometries using three alternative lay-down patterns (0°/90°, 0°/60°/120°, and 0°/45°/90°/135°). The researchers conducted preliminary mechanical and biological analyses to see how the lay-down pattern affected the performances of the fabricated devices. The results demonstrated that the constructions with a 0°/90° pattern gave a compressive modulus that was much higher than those obtained for the other types of scaffolds at a fixed filament diameter, filament spacing, and slice thickness [18].

**Figure 1:**
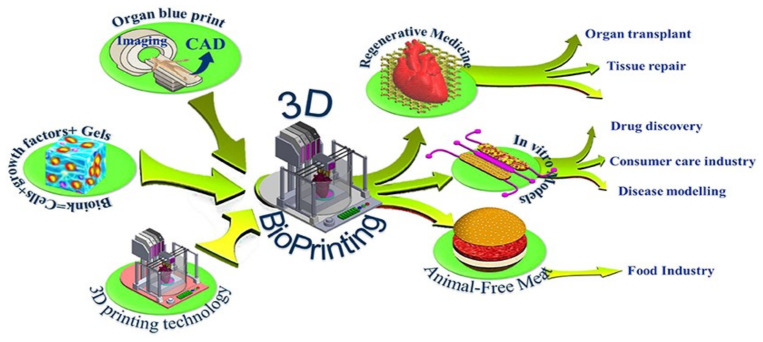
Numerous applications of 3D bio-printing. Reprinted with permission from [20]. Copyright 2021, MDPI. Distributed under Creative Commons Attribution—based license (CCBY 4.0).

For the majority of cases, a three-axis mechanical platform governs the motions of the extruders that print the bio-ink according to the necessary algorithm and shape. The development and application of 3D bio-printing have been steadily rising over the past few years due to certain benefits, including precision deposition, affordability, simplicity, and a controllable cell dispersion. The increase of commercial and scientific efforts as a result of its potential uses has significantly advanced the field. As a result, it is anticipated that the 3D-bio-printing market will be worth USD 1.82 billion by 2022 [19]. Although a lot of focus has been placed on 3D bio-printing in recent years for various applications (shown in Figure 1), various review studies have concentrated independently on crucial elements including fundamentals and procedures. Despite this, there is still potential for improvement, considering the purpose of the technique, the materials employed, and the technology involved. 

The advancement of 3D bio-printing has been the subject of many top-notch review articles. However, there are very few reviews that illustrate 3D bio-printing from all angles, including methodologies, substances, and implementations with the addition of developing hybrid 3D-bio-printing innovations to provide scholars with a more complete description on advancements in 3D bio-printing and potential new orientations for innovation. In this review article, our goal is to briefly outline the fundamental ideas behind 3D bio-printing, highlight some of its applications in the most recent research, and offer some closing thoughts on the tools and methods used in the process. This review will help persuade readers of the importance of common biomaterials in 3D bio-printing as well as the limitations of their use.

## 2. Materials for 3D Bio-Printing

The initial, non-biological uses of 3D printing were for the deposition of metals, ceramics, and thermoplastics. Living cells and biomaterials cannot be processed using increased processing temperatures, organic solvents, or cross-linking agents [20]. Therefore, finding biological materials that are companionable with printing procedures that can also encounter the mechanical and functional requirements for tissue constructs remains the focus [21]. The most crucial aspects for the biomaterials used in 3D bio-printing are biocompatibility, uniformity in disintegration, and printability. Biomaterials are often defined as organic or synthetic materials utilized in biological equipment to mend or even transplant any organ of the body [22]. They are separated into four divisions based on their chemical makeup: metals, polymers, ceramics, and composites. While ceramics and composites have a stronger corrosion resistance [23,24,25] than other classes of materials, metals and composites also have a high mechanical strength. In contrast to other materials, polymers are notable for being biocompatible (BC) and biodegradable [26]. The most practical ingredients for 3D bio-printing are thermoplastic polymers. Nevertheless, they are divided into two primary classes for 3D bio-printing: synthetic polymers and natural polymers (often isolated from animal or human tissues) [27]. Additionally, in the tissue and organ bio-printing industries, the term “bio-ink” is crucial. Biomaterials and living cells are both included in bio-inks, which are created in a cellular matrix. Bio-inks must contain nontoxic, bioactive constituents and have a print temperature lower than physiological temperatures [28] when compared to conventional 3D-printing substances. Both natural and artificial polymers are used in bio-inks because of the substances that are appropriate for them. Bio-inks are being used to create 3D-printed constructions using a broad range of materials (ceramics, hydrogels, elastomers, and polymers) [29]. The highlights and drawbacks of these materials for 3D bio-printing are discussed in this section.

### 2.1. Synthetic Polymers

Synthetic polymers play a noteworthy role as highly applicabile constituents in 3D bio-printing owing to the characteristics they offer, including high robustness, a dominant microstructure, and measured degradability [30]. Chemical synthesis is used to create synthetic polymers, which may be precisely tailored with certain mechanical and chemical properties to match various bio-printing applications. In 3D scaffold construction, poly-glycolic acid (PGA) is regarded as a primary synthetic polymer due to its chemical adaptability, processing simplicity, biocompatibility, and biological characteristics. The glycolic acid monomer created by the biological degradation of PGA is effortlessly eliminated from the physique through certain catabolic routes including carbon dioxide and water [31]. The copolymers of PGA can also retain the mechanical and physical characteristics of PGA. PGA is employed when creating restorable grafts and internal bone-fixation devices. However, PGA’s breakdown products are not hazardous. When PGA’s surface is functionalized via the breakdown of ester bonds, the seeding density and cell spreading can be increased. Additionally, the primary polymer utilized as an antecedent in the FDM process is PLA, a popular, polymeric bio-ink that is a hydrolytically compostable aliphatic polyester with characteristics comprising biocompatibility, process ability, and printing capability [32]. Moreover, one non-toxic polymer with considerable durability is PCL [33]. PCL is a less costly polymer that has admirable BI properties such as rigidity, biocompatibility, and degradability [34]. Its stability typically lasts for six months, with a biological half-life of three years. SLS-printed PCL scaffoldings have properties such as a porous architecture that promotes connectivity, a rough surface, and a bone-like tightness that promotes bone regeneration and cell ingrowth. Nevertheless, the prolonged biological half-life of the scaffold creates an additional barrier in scaffolds designed for uses other than osseous TE. Additionally, the greater hydrophobicity of the substance results in reduced bioactivity, which slows tissue adhesion and cell development [35]. Additionally, a BC, thermoplastic polyester utilized in FDM printing methods is polybutylene terephthalate (PBT) [36]. PBT exhibits high flexibility, simple processing, and allowable strength and resilience. PBT is one of the primary polymers utilized in the biomedical area for in vivo and in vitro biocompatibility. It continues to be useful for printing canine trabecular bone scaffolds and for tissue regeneration. It is also used as a filler in orthopedic surgery. It does not have any distinct benefits and shares the same physical and chemical properties as PCL or PLA. Like most polymers, PBTs will break down in an aqueous medium via oxidation or hydrolysis. Its high melting point (225 °C) and non-biodegradable essence, which results in the production of crystalline residues during in vivo application, restrict its utilization [37]. In musculoskeletal tissue regeneration, filaments manufactured from PLA can be employed to substitute ligaments and non-biodegradable fibers. Poly-D, L-lactic acid (PDLLA) is an amorphous polymer having lactic acid as its primary component. Its inherent hydrophobicity, accessible biocompatibility, and durable mechanical properties make it suitable for biomedical implementations, predominantly in SLA methods. It is one of the polymers that is frequently used to create porous, BC scaffolds. As a result, it is used in tissue engineering and in restorable implants for orthopedic rehabilitation. Another prospective biodegradable elastomer with great biocompatibility and mechanical strength is polyurethane (PU), which has a thermosetting tendency. Water-based PU regular PU can be distinguished by the type of solvent used. The latter uses water as a solvent, whereas the former uses volatile organics [38]. SLA and DLP printing methods use PU, and PLA can be used to assess how well it degrades. The strengths of 3D-bio-printing properties are enhanced by a high printing resolution and good cyto-compatibility. Chondrocyte manufacturing is preferred in cartilage tissue engineering and the creation of bone, muscle, and nerve scaffolds and. It exhibits an optimum elastomeric quality that stands up to repeated contraction and relaxation and is a suitable option for muscle generation [38]. By contrast, an H_2_O-dissolvable polymer—PVA—is utilized in the SLS printing procedure. PVA has a tensile strength comparable to human articular cartilage. With the right adhesive, PVA can build complex structures and offers a suitable matrix for bone-cell ingrowth. Its preferred hydrophilicity and chemical stability enable it to withstand extreme pH and temperature changes, and its semi-crystalline structure ensures that oxygen and other nutrients are efficiently transported to the cell. PVA is frequently utilized in a variety of load-bearing therapies, such as bone-tissue engineering or the repair of craniofacial defects. Due to its water solubility, PVA willfully expands in the presence of water and can be difficult to control [39].

Despite the variety of materials mentioned above, each material has a specific application (this is elaborated on in the “applications” section). We have made an effort to mention a few of the most significant and typical uses of synthetic polymers so that readers are familiar with their uses in bio-printing. The inadequacy of conventional treatments still poses a serious threat to organ regeneration caused by severe losses and/or damages. Nowadays, 3D bio-printing is a noteworthy factor in the quick production of organs as an alternative to conventional techniques. Organ printing begins by employing specific polymers (such as PCL) as a scarified layer that provides strength during printing. The scarified coating is then eliminated by soaking the generated construct in a solution without inflicting suffering to the framework [40]. Recently, researchers attempted to use PU and PCL to apply polymers in the 3D printing of muscle tissue. Due to their adequate firmness and flexible characteristics, many studies have concentrated on developing these materials for printing muscle tissue. These experiments have included a variety of conventional methods, including solvent casting and phase separation, etc. In essence, scaffolds function as essential structural elements in tissue engineering, enabling their incorporation into organ architecture and supplying the required forms. Generalized scaffold fabrication is a requirement for bone-tissue engineering, which includes the steps listed below [41]:

- Selecting the materials for the scaffold and the bone tissue;
- Selecting the cell structure to be used;
- Bio-printing the cells into the scaffoldings;
- Determining the viability of the cells;
- Conducting experiments on animals.

### 2.2. Natural Polymers

Natural polymers, also known as bio-derived resources, are formed from living things and can be removed physically or chemically from their natural environments. Silk, wool, cellulose, and other materials are examples of naturally occurring polymers. These polymers have are used extensively in a variety of commercial sectors, including the food, paper, pharmaceutical, and other industries. Natural polymers that are H_2_O-soluable can dissolve in inorganic solvents that are pleasant to cells, such as cell culture media and phosphate-buffered saline, to produce solutions or hydrogels. Examples of these polymers include gelatin, alginate, fibrinogen, and hyaluronic acid. Natural polymers can be 3D-printed layer-by-layer, employing discrete-stacking rapid prototyping (RP), additive manufacturing (AM), or solid, free-form manufacturing (SFM) principles as their solution or hydrogel states exhibit a certain fluidity [42]. Theoretically, any natural polymer that, under particular circumstances, contains a sol–gel phase transition (i.e., a gelation point) can be printed utilizing a spontaneous, LBL deposition process. In reality, only a very limited number of natural polymers can be printed in films at cell-friendly temperatures (such as room temperature) without the help of the physical, chemical, or biological cross-linking of the included polymer chains. This is due to the fact that only a few naturally occurring polymers can completely satisfy all requirements for the 3D bio-printing of cells, tissues, and organs [43]. Natural polymers have been crucial both during and after the 3D-bio-printing process in a number of ways, including by providing adequate spaces for extracellular matrix (ECM) configurations, biophysical/chemical cues for tissue/organ morphologies, and hierarchical network settings (vascular, neural, and lymphatic). It is possible to successfully and effectively avoid unexpected processing conditions, such as high temperatures, organic solvents, and H_2_O shortages, which have a negative impact on the bioactivities of the compressed cells and/or biomolecules [44]. Numerous natural polymers (such as alginate, chitosan, and decellularized extracellular matrix (dECM)) have been used as the primary ingredient in bio-inks over the past ten years. According to F. Pati et al., bio-inks containing dECM were successfully printed from three tissues [45]. The bio-ink’s dECM compositions, which include traits and biological functions from many tissues, have the ability to closely imitate genuine tissues. In numerous biomedical sectors, these natural polymers have enormous worth for scientific research and extravagant financial profit. These natural polymers for 3D bio-printing have three standout properties:

- Strong biocompatibility;
- Low mechanical strength;
- Quick biodegradability.

Consequently, natural polymers can provide a pleasant and secure situation for cells—especially stem cells—to sprout, relocate, propagate, and/or develop, in contrast to synthetic polymers. The details of a few exemplary natural polymers for 3D bio-printing are discussed in the section that follows.

#### 2.2.1. Gelatin 

**Figure 2:**
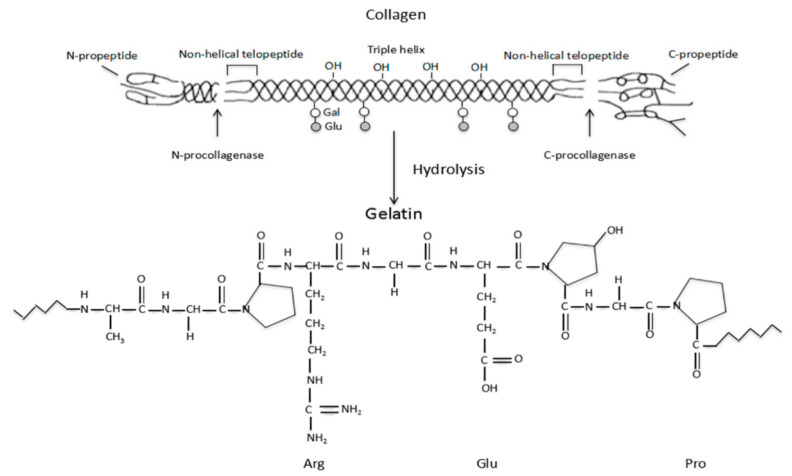
Schematic representation of the collagen hydrolysis to gelatin. Reprinted with permission from [46]. Copyright 2017, MDPI. Distributed under Creative Commons Attribution—based license (CCBY 4.0).

Gelatin is a naturally occurring protein that is generated by the hydrolysis of collagen (shown in Figure 2) [46] and exhibits amphoteric activity with relation to alkaline and acidic amino acid functional moieties. 

Mammalian-derived gelatin has been utilized as a biomaterial for regenerative goals. Due to their good biocompatibility, low immunogenicity, non-cytotoxicity, water solubility, and ability to promote cell adhesion, gelatin and its byproducts have been extensively used in 3D bio-printing [47,48,49]. Gelatin mixture exhibits a peculiar sol–gel transition at 28 °C, which corresponds to the melting point of G hydrogel. Thus, the unique thermal properties of gelati -based solutions enable the injection or extrusion of cells and/or bioactive substances via the plungers of 3D bio-printing, followed by the layering of those substances at relatively tolerable environmental temperatures between 1 and 28 °C. Moreover, gelatin-based solutions and hydrogels are used during and after 3D-printing procedures to sustain the structural performance of the 3D fabricates and to provide spaces for cells and bioactive substances inside the pre-defined 3D constructions. Due to these characteristics, a gelatin hydrogel in the form of gelatin methacryloyl (GelMA) is popular and substantially utilized for DIW printing. Two different types of GelMA for cell-laden bio-printing were evaluated by Lee et al. [50]. They claimed that cell viability had a value of up to 75% in the printed architectures of A and B GelMA. These components’ porous structures and reduced rigidity could help cells survive and proliferate more adequately.

However, naturally gelatin-based hydrogels have two distinct drawbacks in the field of 3D organ-printing [51]:

- Poor mechanical potency;
- Organizational unsteadiness at physiological temperatures (such as 37 °C).

When the printed, unit-filled 3D assemblies are placed in culture media at around 37 °C, it can be observed that the physiological, cross-linked gel phases (or structures) break down quickly. This occurs as a result of the gelatin molecules’ intrinsic cross-linking links disorganizing above the melting point of 28 °C, which compromises the structural performance of the three-dimensional (3D) formations [51]. To put it another way, reversible, physical cross-linking links cause gelled gelati -based constructions to disband instantly in a culture medium. To produce a stable structure [52,53], structures made of gelatin via 3D printing need to be further strengthened.

#### 2.2.2. Alginate 

Brown algae is the source of alginate (commonly known as algin), an anionic polysaccharide. In general, the term “alginate” refers to the salts of alginic acid, which is made up of the building blocks “β-D-mannuronic acid” (M block) and “σ-L-glucuronic acid” (G block) and can signify both the acid itself and all of its derivatives [54]. Divalent cations such as Ca^2+^ (calcium), Sr^2+^ (strontium), and Ba^2+^ (barium) ions can structurally cross-link a material called alginate, which dissolves in water. This property has made alginate exceptionally appealing in the sectors of regenerative medicine (RM), drug delivery, and wound healing [55]. Alginate and composite alginate hydrogels have been enormously utilized as cell-laden bio-inks in numerous 3D-bio-printing methods owing to their low toxicity, non-immunogenicity (excellent biocompatibility), fast degradability, and cross-linkable characteristic (chemical gelling propensity) [56]. Alginate sulfate–nanocellulose bio-inks for cartilage bio-printing implementations were investigated by Michael et al. [57] Alginate Sulfate was mixed with nanocellulose, which has been shown to have excellent printability, to transform it into a printable bio-ink. The results reveal that A-sulfate/nanocellulose ink had worthy printing qualities and that the encapsulated cells’ C II production was stirred by the non-printed BI material. The natural functioning of the cells was greatly influenced by the plunger shape during the printing of the bio-ink [57]. The viscosity of the cell-filled alginate hydrogel, however, is heavily influenced by the density, phenotypic, and molecular weight of the cells throughout the 3D-bio-printing procedures. After chemical crosslinking, cells placed in an alginate hydrogel with an extreme polymer intensity usually have significantly reduced bioactivities. A lower alginate–hydrogel content, meanwhile, allows for greater cell survival and proliferative capacity. Even after chemical cross-linking, however, the mechanical characteristics of the 3D structure rapidly decline when the alginate–hydrogel content is lowered. Therefore, for a standard 3D-bio-printing procedure, an optimal alginate concentration is required to guarantee beneficial cell survival and printing precision [58,59]. 

#### 2.2.3. Collagen

Collagen is one of the BC polymers that has been thoroughly investigated in bioprinting [60]. Throughout the last few years, natural collagen has been enormously exploited as scaffold matter for TE. It is the chief component of musculoskeletal tissue and comprises the ECM of the majority of tissues. In a broad sense, V is a triple-helical, BC protein of biological origin. Therefore, immunological reactions to collagen scaffolding occur infrequently. On porous scaffolds, it can considerably increase osteoblasts’, chondroblasts’, and mesenchymal stem cells’ adherence, multiplication, and development capabilities [61]. Additionally, collagen can improve cellular attachment, adhesion, and proliferation. Nevertheless, it is challenging to 3D-print a collagen solution under ambient circumstances because the characteristics of the low-pH-soluble collagen solution are rapidly affected by hydrogel potential (pH) and temperature. This is because when a solution is neutralized at 37 °C, collagenases and metallo-proteinases can quickly break down collagen strands into amino acids, preventing them from assembling to form a hydrogel. The impact of pH and riboflavin photo-cross-linking on the rheological characteristics and printability of collagen was explored by Diamantides et al. [62]. The findings of their pH analysis demonstrated that the appearance stability of printed fragments throughout the gelation of collagen bio-inks was strongly influenced by pH; however, printability was unaffected by the pace of gelation of collagen bio-inks. In particular, collagen type I and type II have been utilized regularly for 3D-printed scaffoldings for chondro and osseous restoration. Ren et al. concentrated on the bio-printed collagen II hydrogel structures with a gradient chondrocyte compactness for manufactured zonal cartilage. In this investigation, type II collagen played a crucial role in promoting chondrogenic development and the ability to maintain the chondrocyte phenotype. The gradient ECM distribution in the 3D-printed zonal cartilage was favorably linked to chondrocyte density. For better biological effects, the bio-printing technique has modified both cell density and cell dispersion patterns in several zonal areas [63]. The utilization of 3D-printed scaffolds for tissue restoration has three clear benefits:

- In contrast to the conventional tissue-engineering of porous scaffolds, most 3D-printed scaffolds characteristically scale up via networks that are useful for transporting nutrients, oxygen, and metabolites;
- The gradient structural morphology and material composition are advantageous for realizing diverse functions in 3D-printed scaffolds;
- For hard or soft TE, living cells can be directly inserted into biocompatible material.

#### 2.2.4. Hyaluronic Acid

A polymer found in living entities, hyaluronic acid (HA) is made up of d-glucuronic acid and N-acetyl-d-glucosamine [64]. HA has good biocompatibility and biodegradability, which is crucial for cell growth, angiogenesis, and interactions with receptors. Hyaluronidase, β-glucuronidase, and β-N-acetyl-glucosaminidase are enzymes that may quickly break down HA into low-molecular-weight hyaluronic acid and oligosaccharides (i.e., they glycolytically degrade it through a glycolytic pathway) [65]. It is a lubricating hydrophilic polymer that, when added to the previously stated gelatin-based bio-inks, can change viscosity by forming very viscous hydrogels at low concentrations. The limited mechanical properties of HA, as with the majority of natural polymers, lead to a low shape consistency during 3D bio-printing. Although HA has fascinating bioactive qualities and exhibits great biocompatibility for cartilage tissue creation, it lacks the physical characteristics necessary for its use in 3D, extrusion-based bio-printing (EBB). One of these drawbacks is that the material’s solutions lack sufficient viscosity to maintain stability in the reservoir throughout the printing process and, as a result, a uniform, three-dimensional distribution of the cells. Additionally, HA is incapable of gelating, which is necessary to preserve 3D structure after printing [66]. The production of HA formulations appropriate for use as a bio-ink has been made possible by a number of methods based on HA modification [67,68]. However, the majority of these methods have some shortcomings that may restrict their usefulness. Natural gelling agents have drawn a lot of attention in this regard because they do not require any harmful or complicated preparations or gel-forming processes. In this situation, Antich et al. produced highly capable and sustainable bio-printed, 3D hybrid scaffolds for AC restoration using an HA-based bio-ink. Analyzing the mechanical characteristics of the HA-based bio-ink and 3D hybrid construct, it was discovered that HA-based bio-ink increases the production of chondrogenic gene markers—specifically matrix deposition—and tissue development, which enhances cell functionality. These findings point to the bio-printed hybrid scaffold made of PLA and HA-based bio-ink as a suitable candidate for bio-ink that may assist AC formation in vitro [69].

Additionally, many changes have been made to the HA-based 3D-printing techniques to enhance their mechanical characteristics and shape fidelity. The cross-linking of HA with other polymers can be performed physically or chemically. For instance, by employing a UA-light source to photo-chemically cross-link HA and methacrylate, hyaluronic acid methacrylate (HAM) can be formed as a natural/synthetic hybrid polymer [70]. It should be emphasized that although HAMA has improved the poor physical characteristics of HA, the bio-inert characteristics of HAMA, such as the non-biodegradability of polymethacrylate (i.e., PMA), high hardness (or stiffness), and low shape fidelity, have significantly restricted its use in three-dimensional organ-bio-printing areas. As of the present moment, hybrid HAMA–GelMA bio-inks have been used in certain tissue-engineering applications, including the engineering of neuronal, cardiovascular, cartilage, and bone tissues. For instance, Skardal et al. showed that although a low ratio of HAMA/GelMA results in poor mechanical strength but higher cell adhesions, a high ratio of HAMA/GelMA would result in a stiffer structure but a poor cell-adhesive ability. When all factors were considered, the 80/20 HAMA/GelMA ratio was the best option [71]. 

Furthermore, Hauptstein et al. conducted research on hyaluronic acid (HA)-based bio-ink compositions to support 3D bio-printing and improve the quality of deposited, cartilaginous extracellular matrices by the UV-cross-linking of an allyl-modified poly(glycidol) in a range of concentrations [72]. The gels were additionally enhanced with an unmodified, 1 wt.% high-molecular-weight HA (hmHA), and chondrogenic differentiation of the included human mesenchymal stromal cells was evaluated in order to adapt bio-inks to poly(-caprolactone) (PCL)-supported 3D bio-printing. Surprisingly, the addition of hmHA to gels with a modest polymer content (3 wt.%) led to a noticeable improvement in construct quality with uniform ECM distribution across the constructs, independent of the printing procedure. When compared to higher-concentrated constructs (10 wt.%), which exclusively exhibited peri-cellular matrix deposition, the improved ECM dispersion in those constructs was related to a greater construct stiffness during chondrogenic development. This study advances the development of bio-inks by demonstrating the dual functionality of a supplement that supports PCL-supported bio-printing while also enhancing the biological characteristics of the constructs produced. 

#### 2.2.5. Chitosan

The well-known natural polymer chitosan, which is obtained from shrimp shells, is created when chitin is de-acetylated. It is an advantageous choice for tissue engineering due to its biocompatibility, antimicrobial qualities, biodegradability, and inexpensive cost. Lysozymes can biodegrade chitosan to produce amino-sugars [73]. Similar to alginate and HA, the poor mechanical strengths and sluggish gelation characteristics of chitosan solutions have undoubtedly limited their use in 3D organ-printing applications. Chitosan can be physically combined and chemically cross-linked with other supporting polymers, including alginate, gelatin, and collagen, to increase its mechanical qualities as expected [74]. A high chitosan viscosity is typically advised for extrusion-based, hybrid-polymeric hydrogel 3D-bio-printing technologies. Collagen/chitosan, alginate/chitosan, and gelatin/alginate/chitosan have all been widely and recently used as bio-inks in various 3D organ-bio-printing fields. A chitosan hydrogel was employed by Wu et al. [75] to guide cell development. Unusually, chitosan itself can be chemically altered to enhance printability in an acceptable pH range (7–7.4) without compromising its biocompatibility or biodegradability. Gu and colleagues’ research focuses on the proliferation and differentiation of brain cells, and they extruded a mixture of alginate, agarose, and carboxymethyl-chitosan in their experiments [76,77,78]. The mixture of 1.5% alginate and 5% carboxymethyl chitosan (a water-soluble derivative) was first identified as being ideal for print fidelity and the development of human-brain stem cells. The constructs had a depth evaluation of differentiated cell development that reached 169 μm, making them rather thin. In a subsequent work, Gu et al. demonstrated how the scaffold supported the formation of the embryoid body and subsequently controlled differentiation along the neural lineage using induced pluripotent stem cells.

Cheng et al. [79] simultaneously created a composite of chitosan and poly (caprolactone)-diacrylate/poly (ethylene glycol)-diacrylate for 3D printing via photopolymerization. Kingsley et al. (2016) [80] complexed the microspheres with PLL or chitosan using the laser direct-writing technique, in which cells in alginate were expelled by a laser pulse into gelatin/CaCl_2_. By liquefying the alginate core through incubation in sodium citrate, encapsulated cell spheroids that could be shaped into micro-strands were produced. Using chitosan coating as opposed to PLL coating, a breast-cancer cell line (M231) had higher cell viability. Fibroblasts and M231 cells were printed as a single construct to demonstrate their distinct localization and interplay. Despite being a 3D culture, the spheroids or strands in this study were not stacked, and the *z*-axis was about 80 μm. For bone tissue engineering, Lee et al. [81] created scaffolds using chitosan, gelatin, and hydroxyapatite. Demirtaş et al. [82] created a chitosan that exhibited temperature-sensitive gelation and remained in solution up to a pH range between 6.9 and 7 using a mixture of chitosan and glycerol phosphate. Alginate and chitosan–glycerol phosphate were compared with and without hydroxyapatite in printing pre-osteoblasts (MC3T3-E1). A good cell vitality (>90%) was observed across all conditions. Chitosan–hydroxyapatite > chitosan > alginate promotes osteogenesis in MC3T3 cells in accordance with osteogenic gene expression. Chitosan is a feasible substrate for iPSCs, MSCs, neurons, and other cell lines, according to these bio-printing research. They serve as the foundation for investigations that will improve in vitro models and add controlled complexity. Chitosan and polyethylene glycol diacrylate were utilized as bio-inks by Morris et al. and Elviri et al., respectively, to print scaffolds using the stereolithography technique [83,84].

#### 2.2.6. Decellularized Extracellular Matrix

Cell and extracellular matrix (ECM) elements such as collagen, fibronectin, laminin, and glycosaminoglycans are found in tissues and organs [85]. Each tissue and organ has a unique composition that is influenced by interactions between its cells and the ECM. The ECM interacts with and controls the behavior of cells while also being produced by cells [86]. Cell receptors such asintegrins are used by cells to interact with the ECM. Several signaling pathways that are crucial for cellular functioning are activated by cell–ECM interactions. The decellularized extracellular matrix (dECM), on the other hand, is a blend of organic polymers made from various animal tissues including the skin, small intestinal submucosa, and liver [87]. The elimination of cellular components while preserving the natural shape and makeup of the tissue or organ is the true aim of a decellularization (dC) phase. Thus, the final qualities of the created dECM bio-ink are greatly influenced by the chosen dC process. Following dC, the composition and structure of the original tissues may still be largely preserved, which may allow for the creation of tissue-specific microenvironments for the preservation of cell-specific functions. The final components of the dECM can be influenced by the dC processes, which can be either physical, chemical, biological (such as enzymatic), or a mixture of these processes. Chemicals such as acids, bases, detergents, and alcohols have all been utilized in the past, but the only biological techniques that are currently available are enzymes, such as trypsin, and nucleases [88]. Other techniques, such as sonication, heating, exerting pressure, and electroporation, can also be used to decellularize an object [89]. Beyond 15 °C, the resulting dECM-based solutions gel instantly and produce physically cross-linked hydrogels. It was reported that dECM produced from porcine liver could be employed as a useful substrate for hepatocyte cultivation. Through albumin secretion, bile-salt export-pump (BSEP) mRNA expression, and sodium taurocholate co-transporting polypeptide (NTCP)—which was considered a potential scaffold material in3D tissue-bio-printing—the liver-specific dECM could maintain hepatocyte activities [45]. Additionally, dECM can offer cells specialized milieus for the 3D bio-printing of organs. Due to their low viscosity, dECM bio-inks frequently require the assistance of additional supporting polymers in order to achieve basic 3D printability and shape fidelity. For instance, a multi-head tissue-building system has printed an adipose-derived dECM/polycaprolactone (PCL) hydrogel with encapsulated ADSCs, which produced a high cell viability (>90%) [90]. 

The use of dECM-derived bio-inks for various tailored, bio-artificial 3D organ-bio-printing applications is currently gaining popularity in both academic and professional contexts. Some bio-inks made from dECM have been investigated as potential substitutes for clinical uses. However, the degree of mechanical property preservation has a significant impact on clinical outcomes. The ECM of the native tissue or organ is often maintained by dECMs, which provides tissue- or organ-specific microenvironments for cellular growth and function. Growth factors, cytokines, and microRNAs have all been found to be present in a variety of dECM-based scaffolds that have been produced [91,92]. For a transplant to be effective, dECM must meet a number of conditions. After dC, there cannot be more than 50 ng of dsDNA/mg of weight of remaining DNA. Decellularization must be performed gently to prevent harming the ECM and changing its composition. Additionally, it has been widely shown that the decellularized extracellular matrix (dECM) is a bio-instructive scaffold that may drive and modulate cellular responses, such as proliferation and differentiation, as well as aid in vivo tissue repair and regeneration [93]. Additionally, it has been established that cells have a better expansion and differentiation potential when grown on a dECM from their original tissue [93]. Although the precise mechanism is unclear, it is hypothesized that the various tissues’ distinct compositional makeups of the ECM create an environment that is favorable for cell types that are compatible with their respective tissues. This concept gave rise to the idea of using a tissue-specific approach in tissue engineering. For extrusion-based 3D-bio-printing systems, the use of a dECM as an innovative bio-ink has been popular up until recently. The efficient use of dECM bio-inks to create cell-filled, porous adipose, cartilage, and heart tissue, mimicking using a nozzle-based bioprinter, was experimentally verified by Pati et al. [45]. In this instance, when cultured in their corresponding, tissue-matched dECM, bio-inks—particularly in comparison to collagen-I-regulated bio-printed tissues—assisted with high viability and augmented the tissue-specific gene expression of human-adipose-derived stem cells (ADSCs), human turbinate mesenchymal stem cells (hTMSCs), and rat myoblast cells [45]. This further emphasizes the benefits of tissue-specific ECM. Although the use of a dECM provides outstanding benefits for maintaining tissue- or organ-specific functions, it still has numerous other difficulties regarding the 3D bio-printing of complicated organs. First, it is challenging to successfully remove antigenic epitopes that have been produced by the allogeneic or xenogeneic receivers of dECMs. Second, residual DNA or nuclear materials are somewhat preserved in dECMs, which likely influences the behaviors of encapsulated cells. Finally, there are still many issues that need to be resolved in the future, including incredibly bad mechanical qualities, low construction resolution, surprising form-shrinking, and quick degradation rate [94]. 

#### 2.2.7. Other Materials

Several actions are necessary to obtain bio-inks (e.g., increasing the printability). These constraints improve the qualities of the bio-printed materials. Numerous studies have been performed in this area to accomplish this milestone. Making multi-material bio-inks possible is one of the most crucial tactics. A collagen–alginate bio-ink was employed in tissue engineering by Yanga et al. [95]. An alginate–methylcellulose hydrogel was employed by Li et al. [96] as the bio-ink for bio-printing. Additionally, there have been various attempts to use 3D bio-printing to produce silk-based constructions due to the growth in the biological and therapeutic uses of silk. For the creation of soft tissue implants, the inkjet bio-printing of silk–alginate as a bio-ink cross-linked with horseradish peroxidase has been documented [97]. In order to bio-fabricate cardiac tissue constructs, Wang et al. created a fibrin-based composite bio-ink made of fibrinogen, gelatin, HA, and rat ventricular cardiomyocytes at a concentration of 10 × 10^6^ cells/mL. The fibrin was cross-linked by soaking the bio-printed construct in Dulbecco’s modified minimum essential medium (DMEM) containing thrombin for 20 min. Immunostaining was used to confirm the creation of cardiac tissue constructions and showed that the bio-printed constructs responded to medications for the heart [98]. These findings illustrate the use of fibrin bio-inks in regeneration. The study discussed above can be used to produce many materials for 3D bio-printing. In addition to single- and multi-material bio-inks, a novel idea known as the “self-assembling of bio-inks” has emerged for the manufacture of enormous anatomical structures. Numerous studies have shown that self-assembling tissue strands have been used to create nanofibrous hydrogels for bio-printing [99,100].

**Figure 3:**
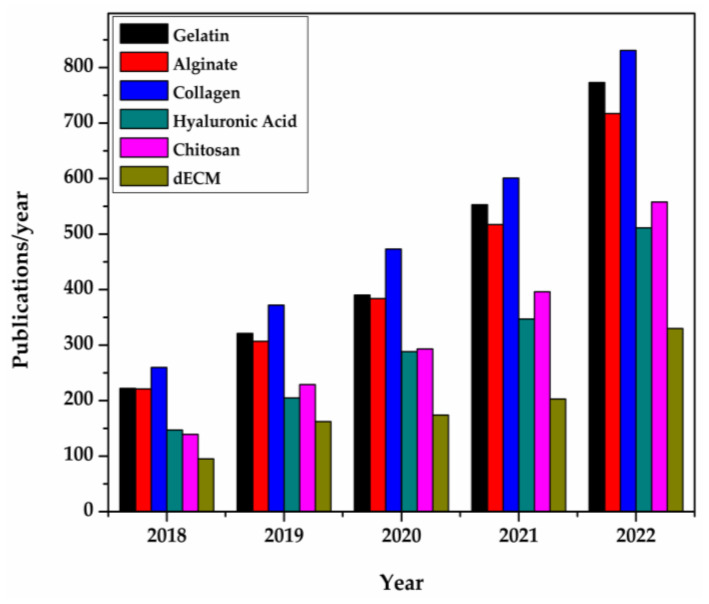
Natural polymer publication/year in 3D bio-printing for tissue engineering [101]. The bars represent the number of publications retrieved from Science Direct. The examined time interval was 2018–2022.

The total number of publications retrieved from Science Direct for the past five years is shown in Figure 3, in which the gradual increase of the use of dECMs is clearly discernible.

Table 1 lists the advantages and disadvantages of a variety of synthetic and natural polymers that are frequently used in various 3D-bio-printing procedures, taking into account the above-mentioned descriptions of the materials that are most frequently used in 3D bio-printing. 

### 2.3. Material Characteristics of Bio-Inks

A complicated manufacturing technique is used to create bio-ink formulations with pre-defined rheological properties in order to 3D-print a rigid or elastic 3D scaffold with evenly spaced components and a controlled shape. As a result, the following qualities are preferred for the bio-inks.

#### 2.3.1. Printability 

One of the most crucial characteristics for a component to be appropriate for 3D bio-printing is its capacity to be effectively used by the printer; in particular, how well the element could be precisely coated with the requisite controllability. This differs amongst printing techniques; therefore, it is challenging to define what printability is. Smooth nozzle dispensing, continuous filament formation with high shape retention, and high structural integrity are all characteristics of bio-inks with good printability. Printability can be impacted by a variety of parameters, including ink composition, viscosity, gelation kinetics, and surface tension. Printing characteristics that can affect the results include extrusion pressure, printing velocity, nozzle diameter, nozzle/printed/ambient temperature, and the printing path. Whatever parameter configurations are chosen, a method of evaluation known as printability is required to describe the structural outcome. Bio-ink printability can be assessed using known techniques such as qualitative description, quantitative analysis, and computer simulation [112]. Ouyang et al. [113] described the structural creation of the pore squared by four filaments in a usual lattice arrangement using a semi-quantitative method. The printability factor (Pr) was defined as the ratio of the ideal square circularity (i.e., *p* = π/4) to the actual pore circularity. An appropriate gelation situation is represented by a lattice structure with a Pr = 1; over-gelation is indicated by a Pr value > 1, and under-gelation is demonstrated by a Pr value < 1. The gelation situation needs a formulation with an acceptable viscosity range and quick transition kinetics from the sol to gel states [114]. For instance, extrusion-based printing can print materials with very high viscosities, whereas inkjet printing has a restriction on material viscosity. However, extrusion-based printing needs the material to have certain inter-layer cross-linking mechanisms or shear-thinning qualities. Bio-inks are anticipated to be shear-thinning liquids with a regulated geometry and porosity that can be extruded in a laminar flow while having adequate mechanical strength to self-support over multiple layers for nozzle-based extrusion bio-printing [115]. Additionally, the viscosity and gel rate of the hydrogel can be changed to increase 3D printability by simply treating the sodium alginate/poly(acrylamide-co-acrylic acid) bio-ink formulation in a solution of pH 14 [116]. It is crucial for the printing material or procedure to shield the cells from this high temperature because some processes demand intense, localized heating of the material for cell deposition. The ability of materials to cushion cells during the deposition process and materials with low thermal conductivity have both been proven to boost cell survivability and biological function after printing. The surface tension between the printing medium and the receiving substrate is another element that significantly affects cell adhesion and development. It is anticipated that the printed material will maintain a vertical tension with the substrate. Before printing, this can be accomplished by covering the substrate with a small layer of substance to increase its hydrophobicity. Rheological characteristics including storage and loss moduli, degree of shear-thinning, and yield stress can also be used to assess bio-ink printability [117,118].

#### 2.3.2. Biocompatibility 

The capacity of a substance to function with a suitable host reaction in a particular situation is referred to as biocompatibility. The primary objective of attaining biocompatibility has evolved over time, shifting from requiring the implantation material to coexist with the host without having any negative local or global consequences to allowing or actively producing positive benefits passively in the host. Additionally, it is anticipated that the material will, in an ideal scenario, allow the cells to replace it with their own ECM proteins that are created at a speed which matches the material’s rate of deterioration after being transplanted into the host and degrading. In a recent study [119], dental implants from mining were subjected to acidic etching and grit-blasting to create surfaces with nanostructures. After four and twelve weeks of implantation, only a very small number of inflammatory cells were observed, demonstrating the biocompatibility of the implants. Additionally, within a few weeks of implantation, the microenvironment within the scaffold was anticipated to promote the growth of blood vessels in or around the implant, creating a favorable environment for the movement of nutrients, oxygen, and waste. Since all byproducts should be harmless, easily digested, and quickly eliminated from the body, the creation of byproducts during the degradation process also defines the biocompatibility of the material.

#### 2.3.3. Gelation

The kinetics of a bio-ink’s gelation or cross-linking are yet another important factor for structural printability. Kinetics control how quickly the deposited bio-ink can cross-link, which has an impact on the shape fidelity of printed constructions. Bio-inks that gel slowly prevent cross-linking from occurring, and the deposited bio-inks spread adversely. Additionally, because of the excessive exposure to gelation stimuli, prolonged gelation time may have an adverse effect on the viability of cells in the constructions (e.g., light, temperature, pH, or other harsh conditions) [120]. However, it is also crucial to manage gelation kinetics because a bio-ink nozzle’s quick cross-linking can result in a sharp shift in the rheological properties and clog the dispensing nozzle. Biomaterials that have been physically cross-linked produce transient networks that are stabilized by weak interactions such as ionic cross-linking, hydrophobic contact, hydrogen bonding, host–guest interactions, and stereo-complex formation [121]. Physical gelation is unsuitable as the only cross-linking method for the solidification of bio-printed constructions due to its dynamic and mechanically fragile nature. As a result, secondary chemical cross-linking is frequently used in conjunction with physical gelation [28]. In contrast to physically cross-linked gels, chemical cross-linking introduces covalent connections into the network, which are believed to increase mechanical characteristics and structural fidelity. Covalently cross-linked inks are often produced using enzyme-mediated cross-linking, photo-polymerization, and click chemistry (e.g., Michael addition and Schiff base formation).

#### 2.3.4. Mechanical Properties 

For the material to maintain a 3D structure following solidification, it must possess enough structural and mechanical qualities. Additionally, a study framework is necessary for cells to adhere to, multiply, and differentiate in an appropriate environment. Additionally, there are considerable impacts on cell adhesion from interactions between cells and the printing medium. From hard, implanted bone to soft tissues such as skin and cartilage, there are varying mechanical needs for materials for different types of tissue engineering. The mechanical qualities are particularly important because the functions of soft tissues primarily depend on such features. To avoid material failure and to reduce fracture under significant strain, the scaffold’s components should establish an effective network-enhancement mechanism [122]. To enable an appropriate load transfer, the mechanical properties of the constructed scaffold should be compatible with those of the host bone. For instance, Ratheesh and colleagues attempted to improve the biomechanical properties of the scaffold by adding 15% *w*/*v* of inorganic bone particles with diameters of 150–500 and 0–500 μm to 10 and 12.5% *w*/*v* methacrylated, gelatin-based bio-inks, respectively [123].

#### 2.3.5. Viscosity

The ability of bio-inks to be printed is greatly influenced by their viscosity; appropriately viscous inks can frequently improve the printing resolution, form retention, and stability. However, high viscosity frequently leads to more shear stress being generated inside the dispensing nozzle, which could harm implanted cells. Therefore, for optimal ink printability, an adjustable bio-ink viscosity is essential. The viscosity of a particular bio-ink formulation is affected by its temperature, shear-thinning characteristics, polymer content, molecular-weight encapsulated cell density, and the addition of rheology-modifying components [124]. Through the use of temperature-controlled nozzles, thermo-responsive biomaterials with temperature-dependent viscosities can be used for 3D bio-printing. A simple method to increase viscosity is to use a high concentration of biomaterials; however, a high polymer density may be hazardous to encapsulated cells and may restrict their access to nutrients and oxygen in a 3D culture system. Depending on the polymer selected, the type of cross-linker used, and the method used to partially cross-link the bio-ink precursor, the polymer’s molecular weight has an impact on the bio-ink viscosity. Recently, the impact of encapsulated cells on the viscosity of bio-ink and the printing quality has been brought to light [125] According to a paper, the viscosity of bio-inks can change after encasing cells. This is likely dependent on the type of cell and the biomaterial. To change the viscosity of ink, rheology-modifying components are frequently used [126]. For instance, Ouyang et al. [127] achieved standard printability for all the examined bio-inks by using gelatin as a universal ingredient to bring thermo-responsive rheology to a variety of photo-cross-linkable hydrogels. In extrusion-based bioprinting, shear-thinning tendencies are advantageous rheological characteristics. The addition of shear-thinning capabilities to bio-inks can be accomplished in a number of ways, including reversible supramolecular and dynamic covalent-bond modifications [128]. Another significant rheological element linked to ink printability is the yield stress. If the applied shear stress is greater than the yield stress, bio-inks behave as a complicated fluid and begin to flow through the printer nozzle [129]. The applied shear stress is removed as the bio-ink exits the nozzle, and the pre-existing yield stress aids in preserving the filament’s form. A bio-ink’s appropriate yield stress also promotes uniform cell distribution in the printed tissue and precludes cell sedimentation in the hydrogel precursor. However, the majority of hydrogels are shear-thinning, which means that as their shear strain increases, so do their viscosities [130]. Some hydrogels also have thixotropic behavior. This means that, when subjected to shear, their viscosity also gradually lowers [131]. Rheological studies of a bio-ink can determine its viscosity, shear-thinning, and thixotropic properties, and numerous mathematical models linking these measures to extrudability and cell injury have been well-established [132]. Rheological characterizations can therefore be utilized to estimate extrudability indirectly. Most significantly, frequency sweeps at varying shear speeds have been used to assess the viscosity of bio-inks. These values can be shown as-is or fitted to the power law equation to determine the consistency index (K) and flow index (n) for comparison between different bio inks. The consistency index relates to the bio-ink’s initial (or zero-shear) viscosity, with lower values indicating increased extrudability. On the other hand, the flow index is related to the shear-thinning properties of the bio-ink, with a flow index of one indicating Newtonian behavior and values closer to zero indicating a higher degree of shear-thinning and, consequently, extrudability [133].

#### 2.3.6. Biodegradability and Surface Characteristics

The printed scaffold is anticipated to deteriorate over time in vivo. In an ideal situation, the pace at which scaffolds degrade and the rate at which the ECM is produced are equal, and the degradation products have no negative consequences on the host. For instance, a waterborne polyurethane scaffold lost 35.62% of its mass after 90 days of deterioration, yet a fluorescence scan revealed that all of the scaffolds had been coated with rabbit chondrocytes after just seven days of incubation [134]. A scaffold transplantation’s effectiveness also depends on the interface between the scaffold and the tissue that needs to be healed, where a variety of responses and interactions occur. As a result, the surface properties of the scaffold, such as its porosity, wettability, and shape, need to be carefully considered. For instance, the first-linked holes should retain a minimum diameter of 100 μm to permit the diffusion of nutrients and oxygen, which are necessary for cell viability, as well as the transfer of waste created by cells. The suggested pore-size range for bone tissue development is between 200 and 350 μm. The scaffold’s surface should be designed to limit cell migration, proliferation, and differentiation. Additionally, the scaffold’s shape is crucial for securing proteins and cells to the surface [135].

The above-mentioned characteristics of bio-inks and consequent scaffolding do not exist independently at any one stage. These qualities are related to one another and may have an impact on one another during the entire process, which includes both physical and chemical changes beginning with the bio-ink in its liquid condition and ending with the formed scaffold and functional implant.

## 3. Fundamentals of 3D Bio-Printing

**Figure 4:**
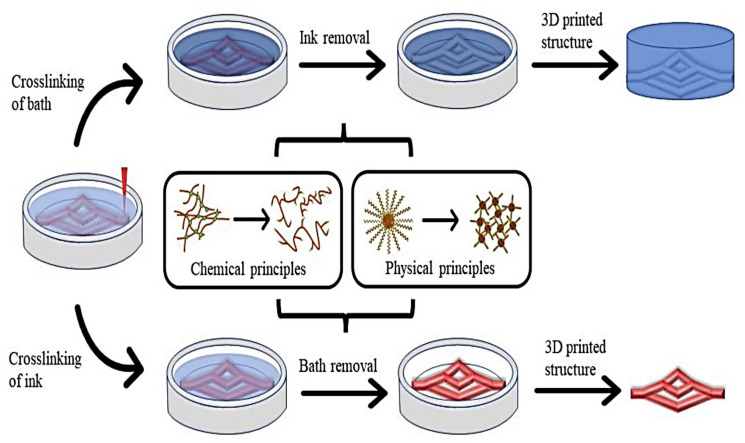
Diagrammatic representation of applications of biomaterials centered on physio-chemical cross-linking principles. Reprinted with permission from [137]. Copyright 2022 MDPI. Distributed under Creative Commons Attribution—based license (CCBY 4.0).

Typically, 3D bio-printing is centered on the LBL exact arrangement of biological components, biochemicals, and biotic cells, by spatial rheostat of the distribution of serviceable components of the generated 3D framework [136], as is represented in Figure 4. 

The term “biomimicry” comes from is an assembly of two Greek words: “bios” (life) and “mimesis” (to imitate). Thus, biomimicry (also known as biomimetics) may be defined as a method of studying nature itself: its structures, methods, and components to be encouraged and energized for the best resolution of human issues. The adhesion, relocation, propagation, and activity of both endogenous and foreign cells are dynamically affected by the inclusion of biomimetic constituents into a bio-printed complex. Materials have a significant impact on how cells attach to surfaces, as well as how big and how round they are, which allows for the regulation of cell proliferation and differentiation in a scaffold [136]. Additionally, nanoscale characteristics such as roughness, notches, and fissures can have an effect on cell adhesion, proliferation, and cytoskeletal organization [138]. Beyond these variables, the 3D environment in a TE structure can further impact cellular morphology and demarcation. A thorough analysis of the natural, tissue-specific components of the tissue of attraction is obligated for a biomimicry process in the 3D bio-printing of components and constructs with specified physiological functions and attributes.

Comparable to embryonic morphogenesis, autonomous self-assembly is a procedure that duplicates a particular organ or tissue in a dish. Extracellular matrix materials and suitable cell signals are produced by the early cellular components of a nascent tissue, and these materials and cell signals help the targeted tissue to organize and arrange on its own. It is possible to use cells for histogenesis via autonomous self-assembly, as well as to modify the makeup, distribution, working, and organizational characteristics of the tissue [139]. To successfully accomplish this, a thorough knowledge of the mechanisms behind embryonic organogenesis as well as the aptitude to control the environmental factors that govern these systems are necessary. The scaffold—namely a 3D, extremely porous base—is crucial for tissue engineering. Cells are multiplied in a culture before being applied to the scaffold. A surface provided by the scaffold allows cells to attach, proliferate, and build the ECM of the fundamental and working proteins and saccharides that give rise to the biotic tissue [139]. The scaffold material’s composition and internal design (the sizes of the arches, facades, apertures, or canals) both affect and regulate the biological characteristics of the cells [140]. Beyond inkjet-based bio-printing (IBBP), the principle is akin to that of traditional inkjet printing (i.e., desktop inkjet printers). Printing is completed without physical touch by precisely dropping tiny droplets of bio-ink onto a hydrogel surface or culture plate [141]. Thermal or piezoelectric actuators can be used for this sort of bio-printing. The extrusion of specific biomaterials, typically prepared as pastes, solutions, or dispersions, is the foundation of the pressure-assisted bio-printing technology [142]. These biomaterials are dispensed using a microscale nozzle orifice (microneedle) to control the movements of pneumatic pressure and plunger- or screw-based pressure in the form of a constant filament onto a static target. After the biomaterial is deposited layer by layer, whole 3D patterns and structures are created. Biomaterials are accumulated onto a substance utilizing a laser as the foundation of energy in laser-assisted bio-printing. A receptive substrate, a ribbon covered with liquid biological ingredients that are coated on the metallic layer, and a pulsed laser source are the typical constituents of this method. The liquid biological materials evaporate as a result of the laser irradiating the ribbon. After the cells have been transferred from the ribbon, the incoming substrate comprises a biopolymer or cell culture setting to sustain cellular adherence and continued expansion [140,143]. 

## 4. Classification of 3D Bio-Printing 

**Figure 5:**
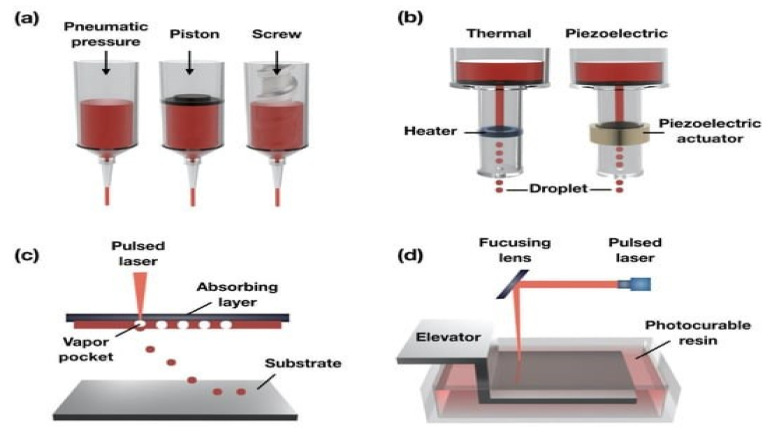
Schematic representation of primary bio-printing methods (**a**) extrusion-based, (**b**) ink-jet, (**c**) laser assisted, and (**d**) stereolithography. Reprinted with permission from [144]. Copyright 2020, MDPI. Distributed under Creative Commons Attribution—based license (CCBY 4.0).

With the proficiency to quickly engineer complex, three-dimensional objects using a top-down method and to enable the uninterrupted accumulation of biotic cells, 3D bio-printing has gained popularity. Inkjet, laser-assisted, and extrusion bio-printing are the three main techniques used for the stacking and structuring of bioactive entities [144], as is shown in Figure 5.

To date, no single bio-printing technique has been able to create synthetic tissues and organs at all bands and levels of complexity. Investigating every aspect of these methods in terms of important elements such as printing resolution, cell viability, and the substance needed is critical for creating the desired 3D frameworks. Different approaches may be used for bio-printing desired tissues depending on their unique guiding principles, material requirements, and a consideration of their benefits and drawbacks [145]. The therapeutic sector and the manufacture of medicinal supplies have undergone significant changes as a result of the fleet rise of 3D bio-printing in the curative field. Complex tissues and organs have been created using this method. Depending on how it functions, 3D bio-printing is divided into various categories [146], discussed below. To achieve the desired tissue production, these procedures can be used separately or in combination.

### 4.1. Droplet-Based Bio-Printing

Droplet-based bioprinting (DBBP) deposits bio-ink in regulated-volume droplets at preset sites. It can be used for a variety of purposes in several areas such as RM, transplantation, high-throughput screening, and cancer, etc. because of its specific switch of accumulation, high resolution, high accuracy, ease of use, and adaptability [147]. There are many droplet-based bio-printing methods, including inkjet bio-printing (refer to Figure 5a), acoustic bio-printing, and others. Similar to the use of 2D, desktop inkjet printers, IBBP is a non-contact technique. It can be categorized as thermal IBBP, electrostatic IBBP, or piezoelectric inkjet bio-printing [148]. The generation of acoustic waves across the BI compartment in piezoelectric inkjet bio-printers uses a piezoelectric actuator [149]. The application of a pressure plate and an electrode produces a voltage pulse that electrostatic inkjet bio-printers use to create droplets. Heat is produced in the BI compartment during thermal inkjet bio-printing, which causes pressure to be created. Despite the fact that the fabrication method is centered on creating ink droplets, the demand for them has grown as a result of their affordable cost, compatibility with living materials, and the rapid building of droplet bio-inks [150]. Owing to their precise regulation over the expulsion of droplets and pico-liter-sized ink droplets, inkjet bio-printing approaches exhibit promise since they offer high-resolution printing [151]. Moreover, biomaterials are protected against damaging stress, such as heat, high voltage, high pressure, and any type of shear stress by the acoustic bio-printing technology. Utilizing a soft acoustic field, droplets are expelled through a nozzle [152]. However, viscous or highly cell-concentrated bio-ink droplets cannot be ejected by mild acoustic waves. There have not been many studies performed on this method. Additionally, micro-valve bio-printing is another droplet-based bio-printing approach that uses an electromechanical valve to regulate the ejection of droplets. The valve that gates the device’s nozzle opening is released from its latch by a voltage-pulse-generated magnetic field. Surface tension is overcome by pressure in the fluid compartment holding the bio-inks, which leads to the formation of a droplet [153]. The small array of pneumatic pressure employed makes this procedure less likely to harm cells than piezoelectric bio-printing. Due to its great spatial resolution, droplet-based bio-printing is thus well-suited to use in the TE and RM methods. These methods also offer reasonable cell survival and decent resolution at a cheaper cost [154]. However, there are also disadvantages to droplet-based techniques. For example, the clogging of the nozzle caused by a too-viscous bio-ink is the main issue.

### 4.2. Laser-Assisted Bio-Printing

Laser-assisted bio-printing (LAB) is a variation on direct-write and laser-induced forward transfer techniques (refer to Figure 5c) [155]. A variety of methods, including laser-guided direct-writing (LGDW), laser-induced forward transfer (LIFT), and modified laser-induced forward transfer (modified-LIFT) processes, are used in laser-assisted bioprinting to deposit cell solutions with high cell concentrations at high speeds (about ≥10 m/s) [156]. Among all the LAB bio-printing methods, laser-induced forward transfer (LIFT) was initially created for writing on metals and has been successfully used to bio-print DNA or organ cells [157]. In order to transfer biological designs to substrates and print biomaterials with greater fidelity, the first LA bio-printer was created in 2004 [158]. In LAB, the contributor has a “ribbon” frame consisting of a layer of BI at the nethermost part and an energy-absorbing layer (EAL) (such as Ti or Au) on the uppermost part. Focused laser pulses from the laser source excite a region on the EAL during printing. Numerous variables, including the laser’s wavelength, power, and pulse duration; the bio-ink layer’s surface tension, thickness, and viscosity; the substrate’s wettability; and the air breach between the “ribbon” configuration and the substrate affect the quality of LAB-printed constructions [159]. In contrast to conventional bio-printing techniques, LAB is a non-interactive and plunger-free bio-printing process. Without the use of a nozzle, LAB is capable of printing a variety of natural substances with elevated viscidness, mammalian cells, and cells with a high density without impairing cell survival or operation. When compared to other bio-printing technologies, LAB offers a considerable advantage because nozzle blockage can be prevented; the suitable bio-ink viscosity range for laser-assisted bio-printing technology is approximately 1–300 mPa s^−1^, which can deliver mammalian cells and has a negligible effect on cell viability and function. Furthermore, the advantages of a laser-assisted bio-printing technique in terms of speed and accuracy are clear given that it can deposit cells in micrometer-resolution at speeds of up to 1600 mms^−1^ and 108 cellsmL^−1^ using a 5 kHz laser-pulse repetition frequency. Additionally, laser-assisted bio-printing technology has a very high cell survival rate (approximately >90%) and has the lowest amount of negative effects on cell proliferation and apoptosis, which can be increased to 100% through specific technical advancements at a significant cost [160]. Moreover, when compared to nozzle-based printing, the laser system’s operation is more complicated, and the characteristics of the “ribbon” cell coating make it difficult to propel cells effectively.

Nevertheless, setting up the “ribbon” configuration for each type of cell or hydrogel takes a while, especially when numerous cell forms are utilized or additional materials are co-accumulated. The negative influences of laser exposure on the cells are not yet comprehended [130]; however, one of the main concerns is the mortality of cells ensuing from thermal destruction in laser-assisted bio-printing (nano-second laser irritation). Hopp et al. [161] employed femto-second lasers to mitigate this damage. While the results were satisfactory, they showed that cell death had accelerated. The number of metal nanoparticles (Ti films with a thickness in the range of 25–400 nm) created during the laser bio-printing process and conveyed in bio-ink microdroplets, on the other hand, has been the subject of research by Zhigarkov et al. The findings show that hardly no nanoparticles are transported into the microdroplet (0.5% to 2.5%) during bio-printing, with the majority remaining in the hydrogel layer on the donor slide. These findings are significant for laser bio-printing applications because the transmitted metal nanoparticles may have an impact on living things. The most important finding of this study was that the concentration of these nanoparticles is too low to have any adverse effects on the printed cells [162]. Laser printing enables the production of spherical nanoparticles with adjustable diameters and offers a potent technique for the exact placement of nanoparticles. Currently, laser printing is used to precisely fabricate metallic (Au, Ag, Al, Cu, and Fe, etc.) and semiconductor (Si and Ge, etc.) nanoparticles with radii that can be adjusted between 50 nm and 1 µm and place them precisely where they are needed on a desired substrate; this has applications in 3D bio-printing. A particularly promising method with many benefits is the 3D laser printing of living cells for the creation of biological tissues and organs. A high printing resolution (<10 µm); high cell survival (up to 100%); high densities of printed cells (>108 cells/mL), comparable with the cell densities observed in living organs; and contamination-free bio-printing at a 2.94 µm laser wavelength, matching the maximal absorption in water, are all possible. The first instance of bio-printing at a 2.94 µm wavelength without the use of an additional absorption layer may be found in [163]. 

However, it is still unknown whether the technology can be scaled up for application in bigger tissues. Laser-assisted bio-printing may be utilized to create cellularized skin constructs, demonstrating the potential of bio-printing for therapeutically relevant cell densities in layered tissue constructs [164]. As a proof of concept, a hole in the calvaria, 3 mm in diameter and 600 mm, was filled with nano-hydroxyapatite to repair a 3D, faulty model of the skull cap in mice. Medical grafts such personalized, acellular, bioabsorbable airway splints that are placed in young patients with localized bronchomalacia have also been created using laser-assisted bio-printing technology [165]. Future functionalities include the possibility of using bio-printing to deliver bio-inks that can be instantly incorporated into the patient’s tissues. On the other hand, using the patient’s own cells might make it easier to apply these kinds of constructs, adding to the structural and functional elements of the tissues.

### 4.3. Extrusion Bio-Printing

Extrusion printing, sometimes referred to as direct-writing, has evolved into one of the best, affordable methods for hasty prototyping thanks to well-known open-source initiatives such as Fab@home and RepRap [166]. EBBP (refer to Figure 5b) is the sole technique that permits for the accumulation of highly viscous substances and dense cell layers to produce three-dimensional (3D) structures. It can be thought of as an expanded application of inkjet bio-printing. Recently, a significant increase in the application of this bio-printing method for tissue engineering and bio-fabrication has been observed [167]. The extruded bio-inks are distributed broadly via two fundamental processes: pneumatical force and mechanical force. Pneumatic force is applied during extrusion using a valve-free or valve-based arrangement of compressed air [168]. A syringe filled with BI is attached to an air pump that has been sterilized. Only the type of bio-inks with shear-thinning capabilities can keep filamentous structure after extrusion because the pneumatic extrusion of the bio-ink creates shear stress. Extrusion without valves is a rather easy process. Valve-based extrusion, however, is favored for high-precision performance. This is one of the most practical methods for printing a bio-ink that contains living cells [169,170]. However, mechanically driven extrusion is appropriate for highly viscid bio-inks such as synthetic and natural polymers. The piston-based extrusion method, which makes use of a piston coupled to an electric motor, is one frequently used mechanical micro-extrusion technique. An electrical pulse that causes the motor to rotate propels the piston frontward, forcing BI out via the outlet. Mechanical processes offer higher resolution and better printability for a wider variety of biomaterials, but they necessitate a more exacting ram and nozzle selection. In actuality, extrusion-based bio-printing is a dispenser system operated by a stage controller positioned on a robotic stage. The bio-inks are applied to a construction material [171], and the piston-propelled accumulation setup controls the overflow of the bio-ink using screw-driven devices. To obtain high-quality results from extrusion bio-printing, it is important to take into account a number of different aspects, including the bio-ink’s viscosity and density, its liquid phase, the extrusion pace, and other exclusive substance-specific characteristics, such as the capacity to cross-link printed strata. Extrusion bio-printing can build components with improved structural support using high-viscosity materials, whereas low-viscosity materials can be used to create environments that are better-suited to preserving cell viability and function [17]. Thus, high cell densities are best achieved using extrusion-based bio-printing, which is also reasonably quick. Printing units at an elevated density is important for application in regenerative drugs as biological tissues already include tightly packed cells. This method can beneficially be applied to a variety of bio-inks. Alginate is one of the most often-used bio-inks for extrusion [172]. EBBP must be relatively viscid to improve resolution. Low cell viability is the result of the large shear forces that arise in pneumatically powered extrusion. Even so, extrusion-based bio-printing cells still have a cell survival of over 90%, despite their resolution being very poor [173,174].

### 4.4. Stereolithography

The stereolithography (SL) technique, based on photocuring, solidifies photosensitive polymers to produce tissue structures under precisely regulated illumination [145]. A UV laser and a focused mirror array are used as the method’s main operating components, shining a ray onto the outer face of the liquid, photocurable resin (refer to Figure 5d). For every strata deposition, the laser moves through a 2D pattern point-by-point while scanning it, and the meticulous beam cooperates with the BI substance to polymerize it in compliance with a specified pattern. The printing pedestal needs to be moved up or down distant from the laser source in order to enable the latest unpolymerized-ink constituent to pass into location for the subsequent strata [175]. The fundamental kinetic parameters of the curing activity, such as the amplitude of the light beam, scanning speed, and period of exposure, regulate the curing time and the thickness of the polymerized surface, which are critical aspects for the performance of the parts produced [176]. The depth of polymerization can also be regulated by adding UV absorbers and photo-initiators to the resin [177]. Visible light SL, on the other hand, is an option that is suddenly being developed in bio-printing through the expansion and maintenance of the edifices, according to Wang et al. [178]. The light-sensitive bio-inks are assisting in the development of the model, as SL bio-printing is designed in such a way that a certain light regulates the production. This is due to the fact that 3D scaffolding designs produced using traditional printing methods are frequently spongy channels with weak characteristics. Consequently, the bio-inks are constructed plane-by-plane [179]. Additionally, SL is renowned for its capacity to print intricate structures with incredibly high-quality internal structures. Van Hede et al. [180] used an in silico model to imitate the expansion of neo-tissue for numerous lattice structures of a 3D-printed hydroxyapatite (HAp) scaffold; from this, it was optimized that an intrinsic, microporous framework with a pore size of 700 μm and a wall thickness of 200 μm spotlighted the augmented bone neoformation in a calvarial rat model. The gyroid, 3D-printed HAp scaffold was created with UV stereolithography using these dimensions as a guide. The gyroid structure that was optimized in silico had a better chance of promoting in vivo bone regeneration than other scaffolds with comparable compositions. As a result, this macroporous, gyroid architecture can support outstanding biological responses and may be a promising design for applications involving the regeneration of intraoral bone [181]. From this experiment, it can also be confirmed that neo-tissue growth acceleration may be anticipated without numerous in vitro and in vivo studies by employing in silico modelling to design the internal structure of a scaffold.

Within addition to its benefits, the SL technique has several drawbacks, such as a high price tag, a moderately sluggish printing speed, and a small range of biocompatible resins that are suited to SL processing. Other issues for medical and hard tissue engineering applications of the SL technique include the inadequate mechanical characteristics of printed scales and the potential cytotoxicity of the uncured resin and residual photo-initiator [182]. Moreover, two-photon lithography is one of many recent advancements in light-assisted bio-printing technologies, and it may provide the highest resolution possible for the bio-fabrication of 3D scaffolds [183]. This method does not require the use of intricate optical systems or photomasks, in contrast to conventional light-assisted systems, in order to print in a photosensitive bio-ink. Unlike the more common UV light sources used in SLA bioprinters, this technique uses laser light; primarily, a near-infrared ultrafast femtosecond laser. Resolution is a crucial indicator of the feature size of the TPL-fabricated nanostructures. Kawata et al. 2001 commented on the difficulties of using two-photon polymerization [184]. They created plastic micro-bulls that were roughly the size of a red blood cell (10 mm in length and 7 mm in height). Since then, numerous techniques for increasing the processing accuracy have been described, some of which involve altering the spatial resolution by modifying the process parameters and by enhancing the photo-initiator qualities. In order to increase the lateral spatial resolution of TPL to 80 nm, Xing et al. evaluated the use of an anthracene derivative (9,10-bis-pentyloxy-2,7-bis(4-dimethylamino-phe-BPDPA)) as a very sensitive and effective photo-initiator, which resulted in a clearer image, brief exposure time, and cross-linking. TPL has been in use for about two decades. However, it has only recently been used for bio-printing applications [185]. In another study, PEG tetra-bi-cyclononyne hydrogels were made into multiscale channels by photodegrading the bio-ink at the desired pattern [186]. They demonstrated a penetration depth of roughly 500 μm and a lateral resolution of roughly 1 μm. Since it enables the production of micro-tissue models with submicron resolution, which could not be easily accomplished with conventional bio-printing techniques, TPL has showed significant potential as a 3D bio-printing technology. Its application in the creation of OoC platforms has yet to be investigated.

### 4.5. Other Printing Techniques

Many investigations have been conducted on the development of 3D bio-printing technologies. The subject of bio-printing has seen the successful development and application of a wide variety of techniques thus far. Nevertheless, other related strategies are also often used in this sector, in addition to the ones that have been discussed. One of these methods was based on magnetic flotation and was known as magnetic bio-printing. Two methods of magnetic bio-printing [187] are used:

- Incubating cells with nanoparticles in an external magnetic field to create gel through electrostatic interactions;
- Combining a label-free cell with a paramagnetic buffer.

Another method using surface acoustic waves is called acoustic bio-printing, which involves depositing cells that are condensed, pico-liter droplets of bio-inks in an acoustic field [188]. Despite numerous investigations using magnetic or acoustic bio-printing, further research is still needed. In tissue engineering/regeneration, bio-plotting is characterized as a 3D-bio-printing process. A syringe is used in versatile rapid prototyping to extrude a material into tubes or spheroids. In this method, UV radiation is used to promote healing as layers of material are deposited on top of one another (layer-by-layer). The choice of material for bio-plotting presents various difficulties, much like acoustic and magnetic bio-printing. This is one of the key methods for creating co-cultured scaffolds, and it does not have to be exact.

**Figure 6:**
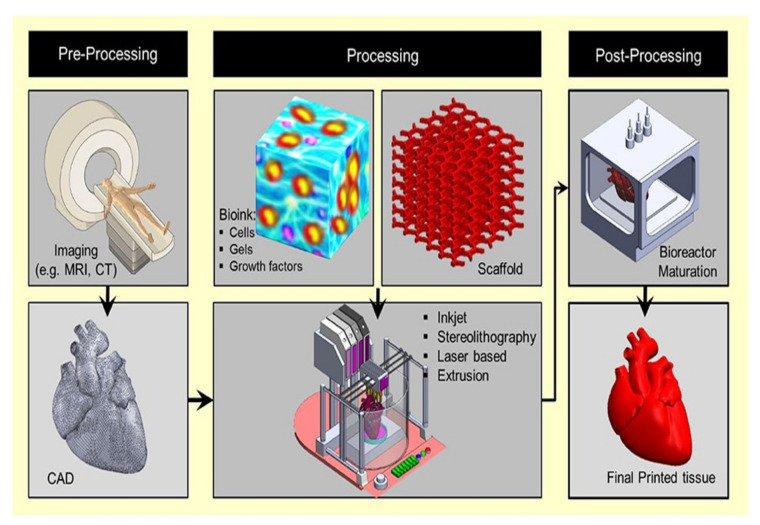
Schematic representation of bio-printing process. Reprinted with permission from [20]. Copyright 2021, MDPI. Distributed under Creative Commons Attribution—based license (CCBY 4.0).

Overall, by utilizing a particular printing technique, the bio-printing equipment creates tissue by combining printing components such as the scaffold, bio-ink, and other additive elements. These processes have varying degrees of precision, stability, and tissue viability. To sustain tissue viability during the maturation phase, the created tissue is then post-processed in a bioreactor to mimic the necessary in vivo environment, shown in Figure 6.

Considering the above-mentioned descriptions of the techniques that are most frequently used in 3D bio-printing, Table 2 shows the benefits and drawbacks [189,190] of a number of techniques that are frequently employed in various 3D-bio-printing procedures.

## 5. Biomedical Applications of 3D Bio-Printing 

The various potential uses of 3D bio-printing are causing it to grow quickly into a significant industry. Numerous studies have recently found the use of 3D bio-printing in biomedical applications to be intriguing, and numerous businesses around the world have helped to further the use of this technology use in medicine by funding research projects in their labs [60,144,169,194]. The capacity to produce the optional product in accordance with particular patient needs makes this technology extremely advantageous for biomedical applications and devices. Bio-printing uses can generally be divided into two categories: (a) tissue regeneration, which includes printing blood vessels, heart valves, musculoskeletal tissues, liver, nerves, and skin; and (b) biomedical applications, which include drug development and drug screening [195]. As a result, the following section of this study will examine current research on the various biomaterials at the cutting edge of bio-printing technology.

### 5.1. Tissue and Organ Regeneration

In order to restore the functional elements of injured tissues and organs, the capacity to regenerate tissue has gained increasing importance. An application of regenerative medicine called tissue engineering seeks to use in vitro and in situ techniques to regenerate certain tissues and restore normal biological functionalities. The implantation of (a) scaffolds alone, (b) cells cultured and other bioactive molecules, or (c) a combined effect of cells embedded within or on scaffolds are the traditional approaches to tissue engineering that support tissue engineering and model the body’s natural extracellular matrix (ECM) [196].

**Figure 7:**
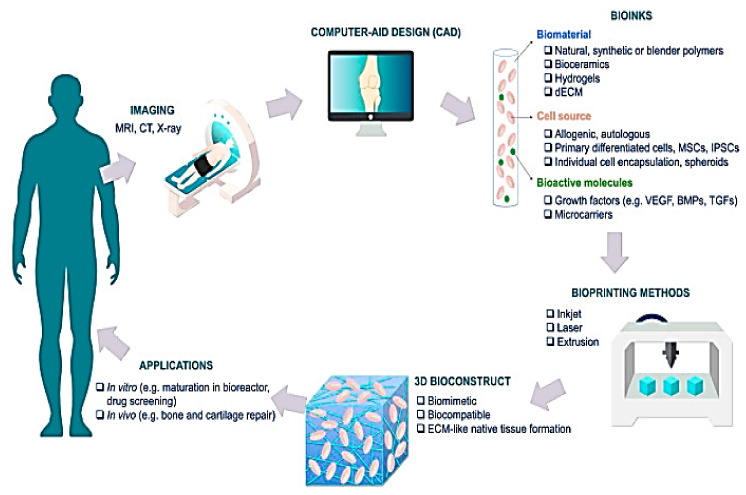
Diagrammatic representation of the bio-printing procedure for various tissue-engineering purposes. Reprinted with permission from [203]. Copyright 2020, MDPI. Distributed under Creative Commons Attribution—based license (CCBY 4.0).

The creation of biocompatible scaffolds that faithfully replicate the native, in vivo environment is the current challenge in the field of tissue engineering. Fundamentally, the topography and architecture of natural scaffolds—specifically, their surface topology and porosity, fiber density, and network structure—determine how cells interact with biomaterials and, consequently, how cells behave [197]. Human tissues that have suffered severe injury or disease need to be treated medically for regeneration or transplantation. The production of tissues and organs for medical purposes, aside from drug testing, is not possible with just the previously available tissue-engineering techniques. Due to a lack of donors and immune reactions, organ transplantation success is also restricted. With regard to 3D bio-printing specifically, it might be challenging to strike a compromise between these biological characteristics and the need for optimum printability. Cell migration, proliferation, and differentiation are significantly influenced by gradients, the ECM organization, and the heterogeneous architectures of natural tissues [198]. The capability of bio-printing technology to fabricate biomaterials for tissue and organ bioengineering has gained significant attention [199,200]. Achieving complex tissue architectures with varied compositions that are well-vascularized, efficient, and reliable is the goal of employing bio-printing in tissue engineering. Computer-aided design/computer-aided manufacturing technologies build the structure of the intended tissue for printing, centered on therapeutic pictures collected from patients [201]. The printed tissues may enable the formation of circulatory networks and offer cells the required behavioral cues [202]. Recent developments in bio-printing-based RM address the repair, substitution, and rejuvenation of impaired or wounded epidermis, neural, osseous, and chondro tissues as is shown in Figure 7. Overall, the reliability of the native morphology, anatomy, porosity, and other characteristics of the regenerated tissue will enhance as a result of the use of 3D bio-printing for tissue regeneration.

#### 5.1.1. Bone

Bone is an intricate mixture of mineral deposits and an organic matrix with a specific structural arrangement. Although bone is a self-healing tissue, its capacity for regeneration is usually rather modest. Mandibular, skull-bolt, and maxillary bone regeneration and restoration can be accomplished quickly and frequently at a low cost using bio-printing, which can accurately duplicate the intricate bone-tissue architecture. An appropriate porous scaffold that has characteristics (biocompatibility and compressive strength, etc.) similar to those of natural bone is needed to rebuild the injured bone because it can support osteoblasts mechanically while they differentiate, proliferate, and create an extracellular matrix (ECM) [204,205]. As a result, the scaffold’s characteristics ought to be comparable to those of the local bone. A resorbable scaffold should have the following desirable characteristics: a porosity of 70–80%, pore size of 300 μm, compressive strength of 5–10 MPa, and an elastic modulus of 20 GPa [206]. Additionally, the ECM is composed of 30% organic and 70% inorganic components. The organic component is composed of 95% type I collagen and 5% non-collagenous proteins, while the inorganic component is made of HAp nanoparticles (50 nm) bound by collagen fibers (5 μm), which provide the bone with a higher tensile strength (150 MPa for cortical bone) [207]. Osteo-conductivity, bio-inertness, and degradability are essential qualities for bio-resorbable materials, but they also need to be biocompatible and not alter the structure or strength of the surrounding bone. 

A method for creating bone tissue was presented by Daly et al. in 2016 [208]. It involved bio-printing a nascent bone-tissue progenitor in vitro and utilizing this constructed rudiment as a blueprint for eventual vascularization and bone formation in vivo. To create the desired framework, researchers employed Ɣ-irradiated A, including Arg-Gly-Asp (RGD) adhesion peptides, conveyed adult mesenchymal stem cells (MSCs), and printed PCL fibers using multiple nozzles. This composite vertebral construction demonstrated noticeably increased amounts of bone growth after twelve weeks in vivo. Vascularized osseous tissue has been created using EBBP and an A scaffold with a good tissue survivability [209]. An A-centered BI with Arg-Gly-Asp adhesion peptides supplemented with polycaprolactone fibers has been used to study whole-bone organ development. A full vertebral body might be created using this group of materials. This vascularized endochondral bone with an accompanying marrow component was supported in vivo, and it may open up the future possibility of bio-printing vertebrae for those who have vertebral osteoporosis or fissures. An extrusion-based, direct-writing bio-printing technique was used by Byambaa et al. [210]. By mixing various gelatin methacryloyl (GelMA) hydrogel bio-inks, they attempted to create micro-structured, bone-like tissue structures that included a perfusable vascular lumen. This method can produce a blueprint for cell-laden manufacturing that will make it easier to mitigate big bone defects. Thus, surface chemistry, wettability, topography, stiffness, porosity, charge generation, and ion leaching are common methods for boosting the activity of the host/biomaterial surface. Different methods are used to modify the active biomaterial and create a particular physical and chemical environment that promotes a positive cellular response in order to change the surface and porosity of the biomaterial. These methods can only be used to create simple shape scaffolds, and their shortcomings include a non-uniform pore distribution, poor porosity, and poorer toughness. The mechanical characteristics of biomaterials are incompatible with those of bone. Bones typically have a network of connected cells, a porosity of 80%, a fracture toughness of 3 MPa, and a compression strength of 5 MPa, etc. Recently, researchers have discovered a number of methods for incorporating biomaterials with superior mechanical strengths and porosities [211]. However, the development of 3D bio-printing technology has shifted the attention to the integration of manufactured tissues into living organisms. Laser-assisted bio-printing was employed by Kérourédan et al. (2019) [212] to create bone tissue in animals with calvarial bone abnormalities. Using LAB, different patterns of endothelial cells, mesenchymal stem cells, collagen, and VEGF may be precisely bio-printed with cell-level resolution into the bone defect. Their findings demonstrate that, under these circumstances, the LAB approach and the integration of VEGF were both secure and well-controlled. At two months, the endothelial cells developed significant organized microvascular networks, indicating that in vivo bioprinting with LAB is an extremely useful tool for pre-vascularizing bone tissue. In addition to pre-vascularization in vitro, in situ construction of pre-vascularized bone tissue with adipose-derived mesenchymal stem cells (ASCs) and HUVECs is also possible. After that, the construct was subcutaneously implanted in immune-deficient mice. Furthermore, mouse pericytes maintained the newly created arteries, showing that the pre-vascularized artificial bone tissue can result in typical bone and vascular growth following implantation [213].

#### 5.1.2. Cartilage

Another area of tissue engineering that receives a significant amount of study attention is the creation of cartilaginous tissues. Collagen and proteoglycans make up the majority of the connective tissue that composes cartilage [214]. Moreover, cartilage tissue has also been 3D bio-printed using thermoplastics such as polycaprolactone (PCL) and PLA. However, cartilage is an avascular tissue (does not naturally repair) with little cell concentration, which makes chondro tissue regeneration challenging. Trophic parameters have been used in cell-packed chondro scaffoldings to alleviate this problem [215]. Through the LA placement of human umbilical-vein endothelial cells (HUVECs), a pre-vascularization technique was created for the quick establishment of sufficient vasculature. More recently, Sun et al. [216] created a dual factor (BMP4 and TGFb3), generating cascade-structured human and rabbit chondro structures with high interconnectedness using mesenchymal stem cells (MSC) and poly (lactic-co-glycolic acid) (PLGA). Overall, bone- and cartilage-tissue bio-printing can offer a feasible substitute for allogeneic or xenogeneic bone grafts. It has even been demonstrated that cartilage TE using alginate and nanocellulose solves the problem of cell adherence, boosting cartilage-ECM deposition [217]. The problem of cell adhesion has also been resolved by merging alginate and collagen, resulting in higher cell survival rates, strength properties, cell growth, and an enhanced capacity for cell attachment [95]. An integrated tissue–organ printer (ITOP) premised on extrusion technology was introduced by Kang et al. [218]. Researchers printed integrated patterns of cell-laden hydrogels and biodegradable polymers attached to sacrificial hydrogels, leaving micro-channels in the tissue constructs to help with nutrition absorption. This method was used to restore skeletal muscle, cartilage, and bone in the mandible and calvaria.

#### 5.1.3. Skin

The application of bio-printing in the building or regeneration of skin has progressively played a significant part in human existence. The exact cell location and the interactions between cells and with the matrix must be considered during the 3D bio-printing process in order to print the skin. Typically, scaffolds used in skin tissue engineering include collagen type I, fibrin, and the synthetic acellular allogeneic dermis. The main cell types for skin printing include keratinocytes, fibroblasts, and stem cells. Skin is one of the complicated, multi-layered organs in the body. To produce a three-dimensional epidermis in this situation, Kim and his team [219] combined the extrusion and inkjet functionalities to develop an entirely new version. By integrating two distinct bio-printing processes, it was discovered that this endeavor was time-efficient. Using a fibrin matrix obtained from human plasma and filled with human fibroblasts and human keratinocytes, Qulez et al. developed skin tissue [220]. In contrast to a commercial graft that dried out and separated from the wound site, bio-printed skin tissue successfully adhered to the wound site in fourteen days, according to a comparative study conducted on mice by Yanez et al. [221]. Albanna et al. [222] revealed the revolutionary, mobile, in-situ design and proof-of-concept evaluation of a skin bio-printing process that allowed for quick, on-site care of extensive wounds despite several studies in skin creation. In this study, scientists printed a bi-layered skin construct made of human fibroblasts and keratinocytes directly onto a full-thickness skin defect on a nude mouse model, demonstrating the bio-printing system’s ability to supply the right cell kinds and concentrations. Three groups of mice, each containing twelve animals, received no treatment, a printed matrix (a solution of fibrinogen and collagen), and a cell-printed matrix (a layer of human fibroblasts topped by a second layer of keratinocytes), respectively. Following the application of sterile gauze and surgical tape, a triple-antibiotic ointment was applied to the wounds in all three groups. A six-week evaluation of the wound area in mice revealed that the cell-printed group experienced a wound closure that was quicker than that of the bio-printed matrix and untreated groups. Overall, when compared to five weeks for both negative controls, the printed skin cells were able to completely seal the incision by three weeks after surgery [222]. The system was then put to the test by delivering allogeneic or autologous dermal fibroblasts and epidermal keratinocytes within a biological hydrogel to a large, full-thickness wound in a porcine model. The outcomes were compared to the outcomes in controls that had either no treatment or a bio-printed matrix alone over an eight-week period. In comparison to the other groups, the in situ bio-printing of autologous cells led to faster wound healing and epithelialization as well as a decrease in wound contraction. A pathologic study confirmed the feasibility of a bio-printed repair by correlating it to the wounds’ outward appearance [222]. Moreover, a 3D bio-printing method that can create a full-thickness skin model with pigmentation was recently published by Min et al. [223]. The dermal layer was created by printing multiple layers of a collagen-hydrogel precursor that contained fibroblasts and cross-linking them with sodium bicarbonate. In order to produce skin pigmentation during a subsequent air–liquid interface culture, melanocytes and keratinocytes were progressively printed on top of the dermal layer. Thus, in terms of the shape and form retention, flexibility, repeatability, and high culture throughput, 3D bio-printing has a number of benefits over conventional skin-engineering techniques [224]. 

Although 3D bio-printing has the potential to design skin, further research is still needed. The resolution, vascularity, ideal cell and scaffold combinations, and cost of bio-printed skin are some of the challenges that must be solved. Before this technology can be utilized in reconstructive surgery, small-scale, 3D skin-tissue models for the toxicity testing of cosmetics and pharmaceuticals, as well as tumor modeling, are expected to be deployed first.

#### 5.1.4. Cardiac and other Tissues

To demonstrate in vitro vascular constructs, researchers have merged generic tissue-engineering procedures with 3D-bio-printing methods. The self-assembly of cells to create vascular constructions, endothelial-cell inkjet bio-printing, and angiogenic growth-factor administration in bio-printed frameworks, etc. are some of these techniques [225]. The bio-printed heart tissue architectures ought to be robust yet pliable, responsive, electro-physiologically reliable, and, most significantly, vascularized in order to support heart function and assist in cardiac tissue regeneration. Norotte and colleagues created a scaffold-free, cell-self-assembly technique for small-diameter vascular regeneration employing human umbilical-vein smooth muscle cells (HUVSMCs), human-skin fibroblasts (HSFs), and porcine aortic smooth-muscle cells (PASMCs). Spatial resolution and arterial wall thickness were two factors that restricted the investigation [226]. Human fetal cardio-myocyte progenitor cells (HCMPCs) were used by Gaetani et al. to exhibit higher cell viability in EBB, bio-printed alginate, and RGD-modified alginate scaffoldings [227]. An endothelialized-myocardium-on-a-chip technique for better vascularization was created by Zhang et al. [228]. Its success suggests a promising future for cardiac transplantation and disease modeling, despite the fact that present tests simply serve as conceptual testimonies. Recently, news of the first “full” heart-bio-printing in the world attracted significant attention. Cells from patients’ omental tissue were adopted by Noor et al. [229] from Tel Aviv University in 2019 so they could be transformed into pluripotent stem cells and developed into cardio-myocytes and endothelial cells. In a suitable anatomical structure and patient-specific biochemical milieu, this research demonstrated the possibilities of building individualized tissues and organs or for pharmaceutical analysis. in addition, 3D bio-printing offers a living valve conduit capable of development and biological integration; it is a better option for treating heart-valve dysfunction. Despite the relatively unique geometry of the heart valve, it is possible to construct each one uniquely using 3D bio-printing technology by taking into account the intricacy of the microstructure of the valve to match the biomechanical and thermodynamic criteria [120]. For the purpose of creating intricate and diverse aortic valve scaffolds, Hockaday et al. proposed a brand-new simultaneous 3D-printing/photo-cross-linking technique. With the aid of poly (ethylene glycol)-diacrylate (PEG-DA) hydrogels enhanced with alginate, native anatomic and axisymmetric aortic valve geometries were 3D-printed. Interstitial cell-seeded scaffolds for porcine aortic valves sustained approximately 100% vitality over the course of 21 days, proving that 3D hydrogel-printing and carefully timed photo-cross-linking can quickly create anatomically heterogeneous valve conduits that encourage cell engraftment [230]. Furthermore, in vitro cardiac tissue was created by Maiullari et al. in 2018 [231] using heterogeneous constructs made of induced, pluripotent-cell (iPSC)-derived cardio-myocytes (iPSC-CMs) and HUVECs. They employed a cutting-edge technique by encasing the cells in a hydrogel made of alginate and PEG-Fibrinogen (PF) and creating unique, high-resolution 3D structures using a microfluidic printing head (MPH). The successful, bio-printed cardiac tissue product contained vessel-like networks that, through in vivo grafting, demonstrated increased efficacy at supporting the integration of the fabricated product with the host’s vasculature. The researchers focused on what they refer to as organ building blocks (OBBs), which consisted of patient-specific induced pluripotent-stem-cell-derived organoids to construct viable cardiac tissue. While preserving high visco-plastic behavior, this quick tissue-construction method encourages self-healing in damaged host tissue [232]. They employed 3D printing to implant perfusable vascular channels, creating a perfusable tissue that could beat synchronously for seven days while receiving nutrition through the perfusable vascular channels. These findings demonstrate that this technique for bio-printing cardiac tissue may closely mirror the behavior of heart tissue while also remaining compatible with pre-printed vasculature [233].

Additionally, the use of bio-printing methods in the restoration of numerous other types of soft tissues and organs has been investigated and are summarized in Table 3. Skeletal muscles and tendons are one instance of this, as they aid in mobility and offer physical integrity [234]. Different research teams have used various biomaterials to generate this type of tissue [235]. Leydig cells of gonad, renal tubular tissues of the kidney, and liver tissues have also been explored for production via bio-printing [236,237]. Additionally, research on the improvement of auricle, nose, and pharynx tissues have been accomplished as well [238]. The quality of visual recovery places restrictions on the human corneal-tissue transplantation techniques now in use. The formation of corneal tissue has been the subject of numerous investigations [239]. Other tissue types being investigated for bio-printing include adipose tissue, lungs, airways, and others [240,241]. The comprehensive investigation into the design of PCL/HA scaffolds for hard tissue regeneration was published by Fucile et al. [242]. In particular, 3D PCL/HA scaffolds with porosities ranging from (50–60°) were created and analyzed using methods based on extrusion/injection techniques, in accordance with an approach already published for the additive production of PCL scaffolds. The potential for creating 3D, customized scaffolds for the regeneration of mandibular defects (such as the symphysis and ramus) was also described as a result of the analysis of the nanocomposite structures at various levels. Given the usages of bio-printing in TE and the regeneration that has been previously discussed, there are many tissue constructs that mimic biological organs, such the pancreatic, neural, cartilage, heart, lung, or muscle tissues. These have all been gradually bio-printed using 3D bio-printing methods.

### 5.2. Drug Delivery and Screening

**Figure 8:**
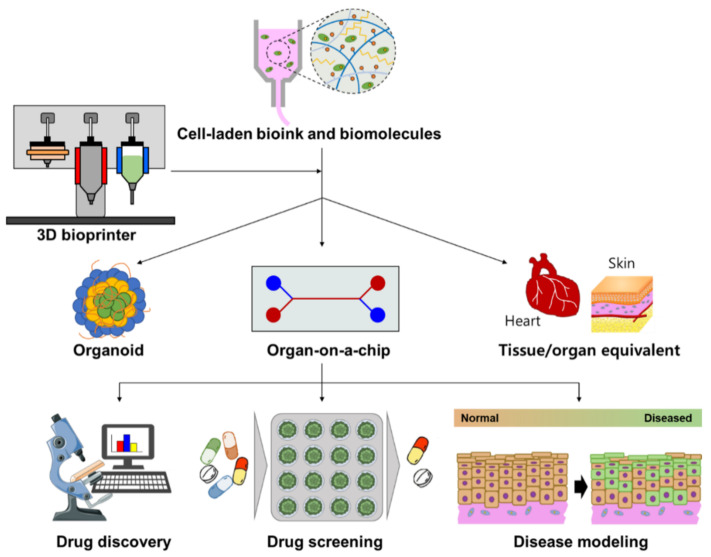
Pharmaceutical applications (drug-discovery, screening, and disease modeling) of 3D bio-printing. Reprinted with permission from [249]. Copyright 2021, MDPI. Distributed under Creative Commons Attribution—based license (CCBY 4.0).

The creation of tissues and organs as well as the provision of signaling pathways and other elements required for the development of blood vessels have all been accomplished using bio-printing innovation in RM, as are illustrated in Figure 8.

As an alternative to traditional oral-medication distribution, a comparable method can be used to deliver pharmaceuticals. A possible new method for creating drug screening devices is 3D bio-printing (shown in Figure 7). For the examination and assessment of the interactions between cells and tested pharmaceuticals, bio-printing offers homogeneous cell distribution on micro-device surfaces in comparison to traditional manual screening procedures [250]. The strategically regulated improvement of the security and effectiveness of drug delivery can be accomplished by affixing to a BM carrier. Natural scaffolds have shown potential in several therapeutic settings as part of 3D bio-printing technologies for flexible medication delivery. Alginate and cellulose are examples of biomaterials that are employed in the pharmaceutical industry as excipients to preserve and safeguard the active medication ingredient, particularly in non-water-soluble pharmaceuticals [251]. Due to its strong biocompatibility and biodegradability, alginate can also be employed as a shipper to restrain and condense medicines, bioactive compounds, proteins, and cells [252]. To develop a liver-specific drug screening system using alginate-encapsulated immortalized hepatocytes, R. Chang and colleagues created a pneumatically-propelled EB bio-printer. This technique imitated the in vivo microhabitats of diverse mammalian tissues and could distinguish the drug metabolism potential useful for assessing the substance of convenience’s effectiveness and toxicity. Additionally, VEGF (vascular endothelial growth factor) has been delivered to hMSCs using a vector made of alginate and polyethylene glycol to promote osteogenic development [253]. IGF-1 was loaded onto neural stem cells (NSCs) using an LBL construction of gelatin and alginate; the alginate allowed for an improved discharge of IGF-1 onto NSCs. The outcome greatly increased NSC differentiation and proliferation, potentially paving the way for a cure for neurological diseases such as a stroke [254]. Additionally, the hydrogels or bio-inks used in bio-printing also have the ability to grip huge quantities of drugs and growth hormones, discharging them progressively to the target site. It is also capable of making customized medications. Drug release is significantly influenced by the cross-linking of the biomaterials [255]. Genina et al. created a printing method for the co-administration of the medications rifampicin and isoniazid. The outcomes revealed an increased treatment efficacy [256].

Bio-printing may be able to distribute growth factors, medications, and gene therapy in an effective and practical manner [257]. In order to investigate potential pharmacological effects on tissues, organs-on-a-chip devices that imitate the routes of typical organ activities can be made via bio-printing [258]. The creation of pharmaceuticals via drug screening and toxicology scrutiny in bio-printed tissue models is made possible by 3D bio-printing, which makes it perfect for simulating human tissues in a way that is as close to natural as possible. Cell types and origin, biomaterials and hydrogels, and printing procedures must be prudently selected in accordance with the original site of drug administration to achieve an appropriate edifice and setting in the TM [259]. Various experimental TMs with various cells, ECMs, and topologies have been constructed to investigate medication efficacy and toxicity. A few of these have also been used commercially. Using IBBP with primary human articular chondrocytes and poly (ethylene glycol) dimethacrylate (PEGDMA) hydrogel, it has been possible to examine the effects of differentiation parameters on chondrogenicity in the creation of cartilage tissue [260]. Furthermore, hMSCs increased levels of angiogenic pointers such CD31 or CD105 expression. The same research team performed additional investigations into ROCK inhibition [261]. For the purpose of testing the toxic effects of the medicines levofloxacin and trovafloxacin, primary hepatocytes and stellate and endothelial cells were utilized to create a scaffold-free hepatic TM [262]. The printed tissue developed micro-capillaries, liver proteins such as albumin, and fibrinogen while maintaining cell viability for up to 42 days. It has been demonstrated that polyethyleneimine alginate nanoparticles are an efficient vector for delivering functional DNA and siRNA into target cells, with practical benefits in the gene suppression of malignant and virally-infected cells [263]. For the sustained systemic discharge of cationic drugs such as imipramine and procaine, nanocellulose components can also be amended with chitosan oligosaccharide [264]. In contrast, the poly-anionic chemical nature of oxidized nanocellulose exhibits micro-porous architectural features with a comprehensive drug-loading aptitude and conveyance [265]. Therefore, employing this technique, vinaigrettes with a latency for regulated and intellectual analgesia and antibacterial release might be created. Moreover, the use of polymer blends as a formulation technique was investigated by Muqdad et al. [266] to address this processability issue and provide programmable drug-release rates from the printed dispersions. The model medication felodipine was effectively made into solid dispersions utilizing FDM 3D printing and polymer mixtures of PEG, PEO, and Tween 80 with either Eudragit E PO or Soluplus. PEG, which has a low melt viscosity, was used to modify the printability of the blends. PEO’s high molecular weight provided the filaments with mechanical flexibility for simple feeding into the FDM printer. Tween 80 was primarily employed as a plasticizer to reduce the processing temperature to safe levels and to resolve the medicine under the investigation’s degradation problems. The good solubilizing qualities of PEG, PEO, and Tween 80 for weakly water-soluble medicines and their plasticizing characteristics for solid dispersion mixes are related to their secondary activities. This study showed how polymer blends could be used to improve pharmaceutical polymers’ poor printability when using FDM 3D printing. The outcomes showed how the intricate interplay between the miscibility of the excipients in the blends, the solubility of the polymer in the media, and the creation of interfaces between printed strips during the FDM printing affected how the dispersions released drugs.

Thus, current studies into the advancement of drug delivery systems for innovative, physiologically active pharmaceutical innovations are expected to produce a plethora of information.

## 6. Pros and Cons of 3D Bio-Printing

The benefits of 3D bio-printing technology have drawn attention to its applications in pharmaceutics, tissue engineering, and healthcare. The research on the bio-printing methodology reveals that important, material-quality characteristics improve with time and that complicated frameworks can be created using this expertise. The production of these assemblies by bio-printing is challenging due to the intricacy of living body systems such as cells, tissues, and organs, each of which has a unique role [267]. However, due to materials made using bio-printing technology’s capacity for biomimicry, these issues can be avoided. With the aid of this technology, structures with various physical and chemical characteristics as well as various functionalities can be created concurrently.

Cell adhesion, growth factor concentration, and the breakdown rate in various biomaterial regions can all be altered through bio-printing. The ultimate objective is to print a full organ that will be physically and functionally comparable in order to end the endless cycle of donor-organ seeking. Bone, skin, cartilage, and tendons are the key organs that will be printed, in addition to the heart [268]. The capacity to place various cell types in various places and the capacity of biocompatible constituents to nearly resemble the miscellany of actual cells are additional benefits of this strategy [269]. The ECM sensitivity and the microenvironment of printed tissue can be matched to that of actual tissues thanks to the utilization of biopolymers and hydrogels as printing resources (PRs) and the nano-structural properties of biotic tissues. However, not all bio-printing techniques may be compatible with the usage of these biocompatible hydrogels and biopolymers as PRs. The majority lack the structural strength needed for the best bio-printing, and issues such as collapse can be observed due to their extremely fragile nature. For instance, the harmful ultraviolet radiation used in SL increases the risk of skin cancer and makes the process laborious and the cell viability short. Ca_3_(PO_4_)_2_ was effectively loaded into gelatin and hyaluronic acid bio-ink by Bishop et al. for bone bio-printing [189]. The ability to customize treatment using the most anatomically appropriate structure possible thanks to 3D-bio-printing technology increases patient compliance [270,271]. Additionally, 3D-bio-printing technology will reduce unnecessary costs by providing rapid and optional production options. 

In addition to many advantages, 3D bio-printing also has certain disadvantages. These restrictions essentially fall into two groups. These issues involve 3D cell and biomaterial manufacturing, post-implantation functionality, and in vivo integration. The best printing material choice is one of the key components of bio-printing technology. These substances need to be appropriate for bio-printing applications. Numerous substances have biological activity, which can lead to unfavorable cellular connections and early or unintended stem cell differentiation. The quantity of cells required for several tissue varieties cannot be reliably provided by current bio-printing technologies [272]. The printing nozzle becoming blocked during production is one of the problems in the engineering progression. Production can also take several hours, depending on the application. To circumvent the nozzle obstruction in these situations, the printing material needs to be uniform and have the right physical qualities for printing. Another difficulty is that, for a successful transplantation process, 3D frameworks must have enough steadiness and mechanical robustness. Particularly when bio-printed TE components are transplanted into the biotic organism, these structures need to enable appropriate cellular nourishment, oxygen delivery to the cells, and in vivo vascularization. Most cells are 100 μm away from capillaries in vivo, which makes it challenging for cells to survive. There are methods to mitigate this issue, such adding angiogenic growth elements to bio-ink to provide vascularization. An artificial vascular system has been suggested in a study for providing nutrients for cells and to eliminate cellular waste [273]. The low bio-printing pace of complicated scale-up assemblies is another prevalent drawback of bio-printing processes, particularly when it comes to multi-material alternative bio-fabrication. For instance, digital light processing (DLP), a technique centered on outer layer prognosis, has a fundamentally more advanced printing resolution and pace than other bio-printing techniques [169]. Furthermore, DLP has a superior consistency and reproducibility in contrast to other techniques for standardizing biological and mechanical features for in vitro TMs, which is a crucial implementation of 3D bio-printing. DLP is a potential bio-printing method that will likely be used in the domain of in vitro modelling within the next several years, despite the fact that it cannot yet match the resourcefulness of EBBP and that scant research involving several resources have been published [273].

## 7. Conclusions and Future Outlook

Bio-printing is a fast-growing field in tissue engineering and RM that aims to replicate the intricate structures of natural tissues and frameworks. The basis for the application of 3D bio-printing in therapeutic and medical settings has undergone significant progress. The most popular and cutting-edge biomaterials used in 3D bio-printing are outlined in this review, along with some of the associated procedures that are frequently taken into consideration by scientists. Furthermore, by presenting the most significant works and placing particular emphasis on highlighting the advantages and disadvantages of 3D bio-printing, an effort has been made to convey the most pertinent biomedical applications of 3D bio-printing techniques, including tissue/organ regeneration and drug-delivery screening. Although there is still a considerable distance to go before we can print an organ, this state-of-the-art technology has demonstrated remarkable properties that will alter the health of the thousands of individuals who die each day while waiting for a donor organ. Many individuals are still wary of implanting a printed organ in a human body. If it is effective, this procedure will resolve a number of concerns, including the organ-rejection problem and the length of the transplant waiting list. It will also fundamentally alter the practice of medicine. There is a great need for research in this area as there are now insufficient biomaterials that can be utilized in 3D bio-printing, even though the technology has the potential to save the lives of many patients who need transplants. Although there has been improvement, 3D bio-printing is still a new and developing technology with enormous potential in manufacturing and healthcare initiatives. For future development to be sustainable, regulation and oversight of 3D bio-printing are also required. Although bio-printed organs are still in their early stages and have shown to be functional in the lab, there is still a long way to go before they can be implanted into real human bodies.

## Figures and Tables

**Table 1:** Overview of pros and cons of common polymers in 3D bio-printing application.

Biomaterials	Pros	Cons	Ref.
Synthetic Polymers			
PGA	Chemical adaptability; processing simplicity; biocompatibility; and biological characteristics.	Bulk erosion resulting in scaffold collapsing, thereby liberating acidic degradation products that affect the body.	[102]
PLA	Biocompatibility; processability; and printing capability.	Releases acidic by-products; brittleness.	[31]
PCL	Less costly; possesses rigidity, biocompatibility, and degradability.	Longer biological half-life develops secondary obstacle in scaffolds; low bioactivity caused by higher hydrophobicity features.	[35,36]
PEG	Good when combined with other components.	Low cell proliferation and adhesion; poor mechanical strength; UV causes cell damage.	[103,104]
PBT	Exhibits high flexibility, simple processing, and allowable strength and resilience.	Breaks down in aqueous media via oxidation or hydrolysis; non-biodegradable essence.	[37,105]
PU	Great biocompatibility, thermosetting tendency, and mechanical strength.	-	[60]
PVA	Hydrophilicity and chemical stability.	Water solubility that bears adversity in controlling.	[39,106]
Natural Polymers			
Alginate	Fast gelation; low cost; good stability.	Poor cell attachment; easily clogs at high concentrations.	[107,108]
Collagen	Promotes cell attachment; good printing abilities; has an RGD sequence.	Poor mechanical stability; slow gelation; easily clogs; soluble in acid.	[36]
Chitosan	Antibacterial and antifungal.	Slow gelation rate; poor mechanical properties.	[109]
Gelatin	Reversible; promotes cell adhesion.	Unstable/fragile; poor abilities without modification; low rigidity; poor shape stability.	[50,110]
Hyaluronic acid (HA)	Promotes proliferation and Angiogenesis; fast gelation.	Rapid degradation; poor mechanical strength and structural stability.	[111]
dECM	Ability to apply materials from the same tissue of interest; the complex biomolecular and physical cues in the ECM are preserved and can support cell growth and viability.	Residual DNA or nuclear materials; poor mechanical qualities; low construction resolution, surprising form-shrinking; quick degradation rate.	[45,94]

**Table 2:** Overview of pros and cons of common techniques in 3D bioprinting application.

Bio-Printing Technique	Pros	Cons	Viscosity & Resolution	Cell Viability	Price	Ref.
Inkjet	High speed; availability; low cost; high efficiency; the capability to bioprint multiple bio-inks at a time.	Lack of precision in droplet placement and size; need for low viscosity bio-ink; heat damage to cell behaviors; difficult to operate and maintain; frequent nozzle clogging.	<15 mPa/s 50–100 μm	>85%	Low	[15,191]
Micro- extrusion	Ability to use high-viscosity bio-ink at the same time and print at high cell density; capability to generate high-freedom degree motion; versatility; cost-effectiveness; user-friendly; sterilization possible.	Distortion of cell structures; low resolution; low printing speed.	<6 $\times \;10^{7}$ mPa/s 100 μm	>45%	Medium	[66,192]
Laser-assisted	High degree of precision and resolution; absence of nozzle; accurate and fast printing; the ability to use high-viscosity bio-ink and print at high cell density.	Complicated preparation process; time consuming; high cost; trace metallic residues; low-flow rate, bio-ink restriction; very high temperature required (up to 1400 °C).	<300 mPa/s 20 μm	>95%	High	[135,149]
Stereolitho-graphy	High degree of fabrication accuracy; low printing time; creation of smooth surfaces.	Use of high-intensity UV light; lengthy post-processing; lack of compatible materials; bio-inks must be photopolymers; utilized photo-cross-linkers are toxic; difficult to bioprint multi-material constructs.	No limitation 100 μm	>90%	Medium	[133,193]

**Table 3:** Overview of applications of 3D bio-printing in tissue engineering.

Tissue/Organ	Polymer	Technique	Cell Source	Outcome	Ref.
Bone	Alginate/PVA	Extrusion Bio-printing	Bone-marrow stem cells	This study demonstrates that bone tissue could be bio-printed using alginate and polyvinyl alcohol bio-inks in appropriate amounts.	[59]
Cartilage	Cellulose/alginate	Extrusion Bio-printing	Human nasal chondrocytes, mesenchymal stem cells	The therapeutic significance and cartilage synthesis in constructs with high fidelity and good mechanical characteristics are revealed in this study.	[243]
Skin	Alginate	Extrusion Bio-printing	Mouse embryonic fibroblasts	The research demonstrates that the PSP-ink employed was non-toxic, and the suggested skin dermis decellularized bio-ink is discovered to be a good contender for tissue engineering applications.	[244]
Heart	Alginate	Extrusion Bio-printing	H9c2 cells, human umbilical-vein endothelial cells	This study reveals that valentine-like constructions with a self-defined height and appropriate mechanical properties may be created utilising 3D bio-printing employing sacrificial and hydrogel materials.	[245]
Vascular Grafts	poly(ethylene glycol) diacrylate	SLA Bio-printing	Human red blood cells	This study reveals the possibility of simultaneous and orthogonal control of tissue architecture and biomaterials for the creation of regenerated tissues.	[246]
Neural tissue	Gelatin methacrylamide	SLA Bio-printing	Mouse neural stem cells	These results demonstrate that, after two weeks of culture, neural stem cells demonstrated neuron differentiation and neurite extension within the printed construct, indicating the 3D-bio-printed neural construct has tremendous promise for regenerating neural tissue.	[247]
Liver	Gelatin methacrylate, glycidyl methacrylate-hyaluronic acid	SLA Bio-printing	Human-induced pluripotent-stem-cell-derived hepatic progenitor cells, human umbilical-vein endothelial cells, adipose-derived stem cells	This study demonstrates that, throughout weeks of in vitro development, the hiPSC-HPCs exhibit phenotypic and functional improvements in the 3D triculture paradigm.	[248]

## Data Availability

Not applicable.

## Abbreviations

3D	Three-Dimensional
AM	Additive Manufacturing
CAD	Computer-Aided Design
dECM	Decellularized Extracellular Matrix
DLP	Digital Light Processing
ECM	Extracellular Matrix
GelMA	Gelatin Methacryloyl
HCMPC	Human Fetal Cardio Myocyte Progenitor Cells
HSFs	Human Skin Fibroblasts
HUVECs	Human Umbilical Vein Endothelial Cells
IBBP	Inkjet-Based Bioprinting
ITOP	Integrated Tissue–Organ Printer
LAB	Laser-Assisted Bioprinting
LBL	Layer-By-Layer
MSC	Mesenchymal Stem Cells
NSCs	Neural Stem Cells
PASMCs	Porcine Aortic Smooth-Muscle Cells
PBT	Polybutylene Terephthalate
PDLLA	Poly-D, L-Lactic Acid
PGA	Poly-Glycolic Acid
PRs	Printing Resources
PU	Polyurethane
RM	Regenrative Medicine
RP	Rapid Prototyping
SFM	Solid Free-form Manufacturing
SL	Stereolithography
TE	Tissue Engineering
TM	Tissue Model
VEGF	Vascular Endothelial Growth Factor
